# Integrative Toxicology Profiling of *Thuja sutchuenensis* Essential Oil: From LD50 Safety Assessment to Hepatic Apoptosis and Gut Microbiota Modulation

**DOI:** 10.1002/fsn3.71296

**Published:** 2025-12-06

**Authors:** Nana Long, Youwei Zuo, Renxiu Yao, Quan Yang, Jian Li, Xiao Zhang, Shiqi You, Yang Peng, Hongping Deng

**Affiliations:** ^1^ Center for Biodiversity Conservation and Utilization, School of Life Sciences Southwest University Chongqing Beibei China; ^2^ School of Laboratory Medicine Chengdu Medical College Chengdu Sichuan China; ^3^ Chongqing Xuebaoshan National Nature Reserve Management Center Chongqing China

**Keywords:** *essential oil*, *hepatorenal effects*, *network toxicology*, *the median lethal dose*, *Thuja sutchuenensis*

## Abstract

In traditional Chinese medicine, the leaves and essential oil of *Thuja sutchuenensis* Franch. are documented in the *Supplement to the Compendium of Materia Medica* for their properties of “cooling blood, detoxifying, and healing wounds”. However, documentation regarding its potential toxicity is lacking. This study aims to evaluate the toxicity of *Thuja sutchuenensis* essential oil (TEO) in mice. The half‐lethal dose of TEO was determined through acute toxicity assessment in mice. Changes in blood and serum parameters were evaluated, and histopathological examination using hematoxylin–eosin staining (HE) was performed to observe the effects of TEO on the liver and kidneys (*n* = 6 per group). A network toxicology workflow identified hepatotoxicity‐related targets and pathways, which were validated by untargeted liver metabolomics and differential analysis with FDR control. 16S rRNA sequencing profiled gut microbiota (alpha diversity, beta diversity). TEO did not elevate systemic inflammatory cytokines (all *p* > 0.05), but decreased leukocytes and increased red cells. HE revealed focal necrosis and mild inflammatory infiltration in the liver of mice, with no notable effects on the kidneys. Network toxicology highlighted PPARG/CYP3A4/TNF axes; enrichment included. Metabolomics detected 611 differential metabolites (log_2_FC ≥ 2, *p* < 0.05); representative changes were Picric acid and Gemfibrozil 1‐O‐beta‐Glucuronide, consistent with oxidative stress. Notably, TEO did not alter the overall composition of the intestinal microbiota in mice but increased the abundance of lactobacilli‐associated taxa, including *Ligilactobacillus*, *Lactobacillus*, and *Limosilactobacillus* (*p* < 0.05). The median lethal dose (LD₅₀) of TEO in mice was found to be approximately 3800 mg/kg. These findings provide a foundation for further studies on the protective effects and potential applications of TEO.

## Graphical abstract

This study provides the first experimental evidence of *Thuja sutchuenensis* essential oil (TEO) toxicity, with an oral LD50 of ~3800 mg/kg in mice. Sub‐LD50 doses cause focal hepatic necrosis via apoptosis/oxidative stress–related pathways without systemic inflammation or renal injury. TEO also enriches Lactobacillus‐related gut taxa while leaving the overall microbiota composition largely unchanged.

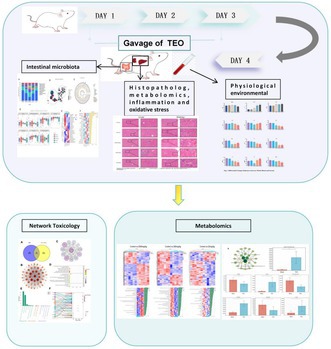

**Abbreviations**
ALB: albumin
ALT: alanine aminotransferase
BP: biological processes
CC: cellular component
Cre: creatinine
DBIL: direct bilirubin
GGT: gamma‐glutamyl transferase
H_2_O_2_
: Hydrogen Peroxide
HE: hematoxylin‐eosin staining
IL‐10: interleukin‐10
IL‐4: interleukin‐4
IL‐6: interleukin‐6
LD_50_
: the half lethal dose
MDA: malondialdehyde
MF: molecular functions
NMDR: non‐monotonic dose–response
NOAEL: no observed adverse effect level
PPI: protein–protein interaction
RNS: reactive nit rogen species
ROS: reactive oxygen species
TBA: total bile acid
TEO: *Thuja sutchuenensis* essential oil
TNF‐α: tumor Necrosis Factor‐alpha
TP: total protein
UA: uric acid
UREA: urea

## 1 Introduction

Belonging to the genus *Thuja* within the Cupressaceae, *Thuja sutchuenensis* Franch. is an evergreen conifer native to China. According to the Compendium of Materia Medica, several Thuja species have long been employed in traditional Chinese medicine to treat conditions such as headache, bronchial mucositis, rheumatism, psoriasis, enuresis, cystitis, and amenorrhea (Ge et al. 2021; Peng and Wang 2008; Tao et al. 2023; Yu et al. 2020). Notably, *Thuja sutchuenensis* has been used as a folk remedy for colds, coughs, and indigestion (Gao et al. 2008). With advances in modern pharmacology, the bioactive constituents of *Thuja sutchuenensis* are now recognized to be predominantly terpenoids, with sesquiterpenes attracting particular attention (Almadiy and Nenaah, 2022; Wang et al. 2020). Extracts from roots, stems, and leaves show strong antifungal and antibacterial activities (Guo et al. 2019; Wang et al. 2022, 2020), and the essential oil exhibits anti‐inflammatory and antitumor effects (Han and Parker 2017; Iwamoto et al. 2001; Kim et al. 2013). These studies underscore the considerable development potential of *Thuja sutchuenensis* essential oil (TEO); however, rigorous safety evaluation (an essential step in chemical development) remains lacking. Essential oils are widely explored as food preservatives, feed additives, and cosmetic ingredients, further highlighting the need for a clear safety profile (Aziz et al. 2018; Lanzerstorfer et al. 2020). However, several essential oils listed as GRAS by the U.S. FDA (21 CFR 182.20) have shown toxic effects at very low concentrations in recent studies, including respiratory impacts, mucosal irritation, acute toxicity, reproductive toxicity, and organ toxicity (Horky et al. 2019; Sandner et al. 2020). Therefore, a scientifically rigorous assessment of acute toxicity is essential when essential oils are intended for therapeutic use or incorporation into cosmetics, foods, or feeds, as such evaluation provides value from multiple regulatory, safety, and translational perspectives.

Based on this, we investigated the effects of orally administered TEO on physiological environments in mice, aiming to provide better evidence for the protection and utilization of *Thuja sutchuenensis*. Toxicological research traditionally includes observations of lethal doses, physiological changes, and organ damage. In recent years, the advent of network toxicology, metabolomics and gut microbiomics has expanded its application fields. This study not only focuses on conventional toxicity indicators but also predicts the hepatic toxicity of TEO, integrating liver metabolomics and gut microbiology to explore its multifaceted effects on the organism. This approach enhances the depth and comprehensiveness of TEO toxicity research.

## 2 Materials and Methods

### 2.1 Preparation of *Thuja sutchuenensis* Essential Oil (TEO)

The leaves of *Thuja sutchuenensis* were obtained from the ex situ conservation base at Xuebao Mountain, Chongqing, China, with full compliance to the Regulations on Access and Benefit‐Sharing of Genetic Resources (ABS) of China (CQNM 02195). Fresh leaves of *Thuja sutchuenensis* (100 g) were hydrodistilled in a Clevenger apparatus (2.0‐L flask) with distilled water (1:10 w/v). Reflux was maintained for 4 h (timed from first distillate), the oil was separated, dried over anhydrous Na_2_SO_4_, yield recorded at 20°C (v/w) (Miya et al. 2021). The density (*ρ*) was determined as 0.8499 g/mL by weighing 1 mL of oil in triplicate and averaging the value. Gas chromatography–mass spectrometry (GC–MS) analysis (Agilent 5975B) employed a DB‐5MS column (30 mm × 0.25 mm i.d., 0.25 μm) with injector/interface temperatures at 250/290°C. The GC oven was initially set to 70°C, then ramped at 5°C/min to 290°C and held isothermally for 10 min. High‐purity helium (≥ 99.999%) was used as the carrier gas at a constant flow of 1.0 mL/min. Essential oil samples (10 mg in 1 mL diethyl ether) were injected in pulsed split mode: an initial inlet flow of 1.5 mL/min was applied for 0.5 min, then reduced to 1.0 mL/min with a 40:1 split ratio for the remainder of the run. The mass spectrometer operated in electron ionization (EI) at 70 eV, scanning m/z 35–650 at 0.34 s per scan. Compounds were identified by comparing retention indices (RI) calibrated with n‐alkanes (C_7_–C_40_)‐and mass spectra against the Wiley 6, NIST11, MassFinder 2.3 databases and an in‐house MS library (Table 1) (Long et al. 2025). For animal dosing, TEO was emulsified with Tween 80 to a final concentration of 5% v/v (42.50 mg/mL) under ultrasonic agitation.

**TABLE 1:** Analysis of essential oil of *Thuja sutchuenensis*.

NO	RT	Area Pct	Library	MF	Ref	CAS	Qual
1	3.813	0.2813	p‐Xylene	C_8_H_10_	5631	106‐42‐3	95
2	5.3589	33.3779	γ‐Terpinene	C_10_H_16_	17,501	99‐85‐4	90
3	5.7149	3.1011	3‐Carene	C_10_H_16_	17,457	13466‐78‐9	91
4	6.7463	1.7858	β‐pinene	C_10_H_16_	17,719	18172‐67‐3	97
5	7.3266	7.0674	β‐Myrcene	C_10_H_16_	17,490	123‐35‐3	96
6	9.2372	2.4024	D‐Limonene	C_10_H_16_	17,468	5989‐27‐5	99
7	9.805	0.0994	trans‐β‐Ocimene	C_10_H_16_	17,523	3779‐61‐1	93
8	10.4796	0.0129	β‐Ocimene	C_10_H_16_	17,485	13877‐91‐3	95
9	12.9332	0.1985	2‐Nonanone	C_9_H_18_O	22,228	821‐55‐6	91
10	13.4666	3.0748	Linalool	C_10_H_18_O	29,730	78‐70‐6	97
11	14.3915	0.0707	(E)‐para‐2‐menthen‐1‐ol	C_10_H_18_O	30,100	29803‐81‐4	93
12	14.7275	0.0215	1,4‐Hexadiene, 5‐methyl‐3‐(1‐methylethylidene)—	C10H16	17,671	113687‐24‐4	95
13	15.2602	0.341	(+)‐2‐Bornanone	C_10_H_16_O	28,038	464‐49‐3	97
14	15.8664	0.0497	n‐Amylbenzene	C_11_H_16_	25,418	538‐68‐1	93
15	16.3263	0.1416	endo‐Borneol	C_10_H_18_O	29,771	507‐70‐0	97
16	16.4436	0.0987	α‐phellandren‐8‐ol	C_10_H_16_O	28,061	1686‐20‐0	86
17	16.7744	0.4846	Terpinen‐4‐ol	C_10_H_18_O	29,794	562‐74‐3	96
18	17.3717	0.6039	(+)‐α‐terpineol	C_10_H_18_O	30,147	7785‐53‐7	90
19	17.9409	0.0736	(‐)‐Verbenone	C_10_H_14_O	26,717	1196‐01‐6	98
20	18.3181	2.9793	Fenchyl acetate	C_12_H_20_O_2_	66,894	13851‐11‐1	99
21	18.7694	0.1193	Citronellol	C_10_H_20_O	31,575	106‐22‐9	98
22	18.8572	0.0588	Bicyclo[3.1.1]hept‐2‐en‐6‐ol, 2,7,7‐trimethyl‐, acetate, [1S‐(1.alpha, 5.alpha, 6.beta)]—	C_12_H_20_O_3_	64,905	50764‐55‐1	87
23	19.6085	0.0584	Linalyl acetate	C_12_H_20_O_2_	66,887	115‐95‐7	91
24	19.6606	0.084	Geraniol	C_10_H_18_O	29,729	106‐24‐1	96
25	19.7834	0.0687	6‐Octenoic acid, 3,7‐dimethyl‐, methyl ester	C_11_H_20_O_2_	55,451	2270‐60‐2	95
26	20.1424	0.0484	Geranial	C_10_H_16_O	28,159	141‐27‐5	91
27	20.3388	0.108	(Z)‐Undec‐6‐en‐2‐one	C11H20O	41,321	107853‐70‐3	95
28	20.5797	2.4582	Bicyclo[2.2.1]heptan‐2‐ol, 1,7,7‐trimethyl‐, acetate, (1S‐endo)—	C_12_H_20_O_2_	67,036	5655‐61‐8	99
29	20.8713	0.0919	2‐Undecanone	C_11_H_22_O	43,210	112‐12‐9	96
30	21.0343	0.0505	trans‐Pinocarvyl acetate	C_12_H_18_O_2_	64,731	1686‐15‐3	83
31	21.6221	0.0972	Phthalic anhydride	C_8_H_4_O_3_	25,847	85‐44‐9	95
32	21.808	0.0306	Decanoic acid, methyl ester	C_11_H_22_O_2_	57,296	110‐42‐9	86
33	22.3384	0.0096	1H‐Indene‐4‐carboxaldehyde, 2,3‐dihydro—	C_10_H_10_O	24,097	51932‐70‐8	91
34	22.5552	0.4797	1,3‐Cyclohexadiene, 1‐methyl‐4‐(1‐methylethyl)—	C_10_H_16_	17,678	99‐86‐5	94
35	22.6838	0.1456	2,4,6‐Cycloheptatrien‐1‐one	C_7_H_6_O	5627	539‐80‐0	93
36	22.9905	0.903	β‐Bisabolene	C_15_H_24_	74,662	495‐61‐4	93
37	23.3117	0.0449	Copaene	C_15_H_24_	74,561	3856‐25‐5	98
38	23.4286	0.2044	α‐Amorphene	C_15_H_24_	74,835	483‐75‐0	89
39	23.5437	0.2151	Geranyl acetate	C_12_H_20_O_2_	66,885	105‐87‐3	91
40	23.7192	0.0623	γ‐Muurolene	C_15_H_24_	74,670	30021‐74‐0	99
41	23.8015	0.2009	β‐Elemene	C_15_H_24_	74,940	515‐13‐9	91
42	23.9956	0.0791	Aromandendrene	C_15_H_24_	74,618	489‐39‐4	98
43	24.0693	0.0539	Di‐epi‐α‐cedrene	C_15_H_24_	74,706	50894‐66‐1	93
44	24.1662	0.7458	Longifolene	C_15_H_24_	74,595	475‐20‐7	99
45	24.4161	0.1408	(1R,5R)‐1‐Isopropyl‐8‐methyl‐4‐methylenespiro[4.5]dec‐7‐ene	C_15_H_24_	74,791	55732‐78‐0	86
46	24.5724	2.0254	Caryophyllene	C_15_H_24_	74,603	87‐44‐5	99
47	24.7056	0.0723	3, 5, 5, 9‐Tetramethyl‐4a, 5, 6, 7, 8, 9‐hexahydro‐2H‐benzo[7]annulene	C_15_H_24_	74,804	1000412‐94‐8	87
48	24.8758	0.2879	cis‐Thujopsene	C_15_H_24_	74,623	470‐40‐6	99
49	25.2532	0.9512	Paeonol	C_9_H_10_O_3_	40,302	552‐41‐0	95
50	25.3652	0.4402	α‐himachalene	C_15_H_24_	74,937	3853‐83‐6	99
51	25.5049	1.307	1,4,7,‐Cycloundecatriene, 1,5,9,9‐tetramethyl‐, Z,Z,Z—	C_15_H_24_	74,761	1000062‐61‐9	98
52	25.5731	1.07	Dimethyl phthalate	C_10_H_10_O_4_	64,177	131‐11‐3	95
53	25.839	0.2852	(1R,4R,5S)‐1,8‐Dimethyl‐4‐(prop‐1‐en‐2‐yl)spiro[4.5]dec‐7‐ene	C_15_H_24_	74,799	729602‐94‐2	98
54	26.0591	0.1323	Amorphadiene	C_15_H_24_	74,926	92692‐39‐2	93
55	26.1456	0.5088	γ‐Himachalene	C_15_H_24_	74,879	53111‐25‐4	92
56	26.258	0.8387	α‐Curcumene	C_15_H_22_	72,773	644‐30‐4	99
57	26.3722	0.3132	β‐Selinene	C_15_H_24_	74,958	17066‐67‐0	99
58	26.5667	0.7608	(‐)‐α‐Cedrene	C_15_H_24_	75,004	469‐61‐4	93
59	26.721	0.3693	β‐Himachalene	C_15_H_24_	74,857	1461‐03‐6	99
60	27.0426	0.561	Butylated Hydroxytoluene	C_15_H_24_O	91,358	128‐37‐0	98
61	27.1622	0.0511	Bicyclo[4.4.0]dec‐1‐ene, 2‐isopropyl‐5‐methyl‐9‐methylene—	C_15_H_24_	74,782	150320‐52‐8	91
62	27.2951	0.8934	β‐Sesquiphellandrene	C_15_H_24_	74,824	20307‐83‐9	98
63	27.4642	0.0414	(E)‐1‐Methyl‐4‐ (6‐methylhept‐5‐en‐2‐ylidene)cyclohex‐1‐ene	C_15_H_24_	74,778	53585‐13‐0	93
64	27.669	0.0168	α‐Muurolene	C_15_H_24_	74,668	10208‐80‐7	90
65	27.7362	0.0466	(E)‐α‐bisabolene	C_15_H_24_	74,803	25532‐79‐0	93
66	27.8196	0.0908	α‐Calacorene	C_15_H_20_	71,052	21391‐99‐1	98
67	28.2962	0.2064	Nerolidol	C_15_H_26_O	93,705	142‐50‐7	90
68	28.4516	0.0419	Globulol	C_15_H_26_O	93,714	489‐41‐8	80
69	28.7264	0.3251	Spathulenol	C_15_H_24_O	91,515	6750‐60‐3	99
70	28.8091	0.6608	Caryophyllene oxide	C_15_H_24_O	91,338	1139‐30‐6	94
71	29.0399	0.1419	(3R,3aR,5S,6R,7aR)‐3,6,7,7‐Tetramethyloctahydro‐3a,6‐ethanoinden‐5‐ol	C_15_H_26_O	93,827	50657‐30‐2	94
72	29.2214	0.9812	Longiborneol	C_11_H_9_NO_3_S	93,880	465‐24‐7	99
73	29.3142	0.2373	Cedrol	C_15_H_26_O	93,690	77‐53‐2	96
74	29.4445	0.3678	Humulene epoxide ii	C_15_H_24_O	91,455	19888‐34‐7	99
75	29.6753	0.0955	(+)‐ledene	C_15_H_24_	75,023	21747‐46‐6	89
76	29.8335	0.0916	α‐Elemene	C_15_H_24_	74,896	5951‐67‐7	96
77	30.1273	0.1488	(−)‐Spathulenol	C_15_H_24_O	91,306	77171‐55‐2	95
78	30.2109	0.0948	tau‐Cadinol	C_15_H_26_O	93,720	01‐11‐5937	96
79	30.4515	0.8441	3‐Butylisobenzofuran‐1 (3H)‐one	C_12_H_14_O_2_	60,757	6066‐49‐5	95
80	30.8091	0.0733	7‐epi‐cis‐sesquisabinene hydrate	C_15_H_26_O	93,766	1000374‐17‐9	87
81	30.9389	1.231	Z‐Butylidenephthalide	C_12_H_12_O_2_	59,093	72917‐31‐8	98
82	31.3559	0.0908	(±)‐Dictyopterene A	C_11_H_18_	26,814	22822‐99‐7	90
83	32.0237	0.8522	Senkyunolide	C_12_H_16_O_2_	62,518	63038‐10‐8	94
84	32.1513	0.2976	Neocnidilide	C_12_H_18_O_2_	64,871	4567‐33‐3	94
85	32.4096	5.7084	(E)‐Ligustilide	C_12_H_14_O_2_	60,851	81944‐08‐3	99
86	33.0906	0.0759	Cyclopentadecane	C_15_H_30_	81,273	295‐48‐7	95
87	35.074	0.0371	1‐Formyl‐2,2‐dimethyl‐3‐trans‐(3‐methyl‐but‐2‐enyl)‐6‐methylidene‐cyclohexane	C15H24O	91,474	1000144‐09‐7	83
88	35.638	0.1011	Rimuene	C_20_H_32_	146,645	1686‐67‐5	99
89	36.0701	0.0548	Methyl palmitate	C_17_H_34_O_2_	144,202	112‐39‐0	98
90	36.2955	0.0456	(‐)‐15‐Beyerene	C_20_H_32_	146,582	2359‐73‐1	98
91	36.6303	0.0522	m‐Camphorene	C_20_H_32_	146,589	20016‐73‐3	99
92	36.7133	0.0279	(E)‐1‐(6,10‐Dimethylundeca‐5,9‐dien‐2‐yl)‐4‐methylbenzene	C_20_H_30_	144,404	55968‐43‐9	92
93	36.9199	0.1898	n‐Hexadecanoic acid	C_16_H_32_O_2_	129,145	00057‐10‐3	99
94	37.3025	0.0299	p‐Camphorene	C_20_H_32_	146,590	20016‐72‐2	98
95	37.393	0.0429	Ethyl palmitate	C_18_H_36_O_2_	159,424	628‐97‐7	93
96	38.1329	0.1648	Senkyunolide H	C12H16O4	95,394	94596‐27‐7	99
97	38.7307	0.0431	7‐Isopropyl‐1,1,4a‐trimethyl‐1,2,3,4,4a,9,10,10a‐octahydrophenanthrene	C_20_H_30_	144,408	109680‐01‐5	99
98	39.3893	0.0226	trans‐13‐Octadecenoic acid, methyl ester	C_19_H_36_O_2_	172,356	1000333‐61‐3	97
99	39.8614	0.0084	Methyl stearate	C_19_H_38_O_2_	174,782	112‐61‐8	97
100	40.1071	0.1275	Linoleic acid	C_18_H_32_O_2_	154,669	60‐33‐3	99
101	40.3638	0.0237	7‐Pentadecyne	C15H28	79,246	22089‐89‐0	80
102	40.4889	0.0204	Linoleic acid ethyl ester	C_20_H_36_O_2_	185,429	544‐35‐4	99
103	40.62	0.0408	13‐Tetradecen‐1‐ol acetate	C_16_H_32_O_3_	126,822	56221‐91‐1	90
104	42.0539	0.0812	Ent‐beyer‐15‐en‐18‐ol	C_20_H_32_O	163,791	20107‐90‐8	86
105	42.2961	0.0023	Monoolein	C_21_H_40_O_4_	234,104	111‐03‐5	83
106	42.4033	0.0027	2‐Heptadecenal	C17H32O	124,867	1000143‐48‐6	90
107	42.8272	0.0338	Totarol	C_20_H_30_O	161,805	511‐15‐9	91
108	43.5722	0.0141	Ferruginol	C_20_H_30_O	161,789	514‐62‐5	97
109	43.8788	0.0012	2,4‐dimethylbenzo[h]quinoline	C_15_H_13_N	78,098	605‐67‐4	87
110	47.3062	0.0238	1,4‐Bis(trimethylsilyl)benzene	C_12_H_22_Si_2_	93,144	13183‐70‐5	83

### 2.2 Acute Toxicity Assessment

The acute toxicity of the oil was evaluated according to the modified Lorke's method (Lorke 1983; Miya et al. 2021), the acute toxicity should be tested in 2 phases (fixed doses of 10, 100, and 1000 mg/kg in phase I; 1600, 2900, and 5000 mg/kg in phase II; *n* = 3/step). Mortality was monitored at 30‐min intervals for 24 h. The LD_50_ was estimated as the geometric mean of the lowest dose causing death and the highest dose causing no death as described by Lorke and subsequent methodological descriptions (Chinedu et al. 2013; Lorke 1983; Miya et al. 2021). Animal experiments were performed according to ARRIVE guidelines and licensed by Chengdu Medical College IACUC (IACUC‐24‐068).

### 2.3 Group and Administration

As detailed earlier, the acute toxicity screen (modified Lorke's method) yielded an oral LD₅₀ of approximately 3800 mg/kg, informing the upper bound of our testing window. We therefore adopted log‐spaced dosing to capture sublethal effects while enabling dose–response analyzes. Healthy adult Kunming mice (18–22 g) were randomized into five groups (*n* = 10 per group; equal males and females): physiological saline (vehicle control), 0.01% Tween‐80 (solvent control), and TEO at 2000 mg/kg (high), 200 mg/kg (medium), or 20 mg/kg (low). All treatments were administered via oral gavage once daily for three consecutive days. The experiment terminated 24 h after the final administration. The body weight of the mice was measured before the experiment began and after its completion. After the observation period, the mice were anesthetized with 3% isoflurane (in 100% O₂ at 3 L/min, RWD Life Science Co. LTD, China), followed by blood collection via orbital puncture and euthanasia by cervical dislocation.

### 2.4 Physiological Environmental Changes

After the experiment, blood was collected from the orbital sinus. EDTA‐anticoagulated whole blood was analyzed using an automated hematology analyzer (DS‐261; Jiangsu Sinnowa Medical Technology Co. Ltd., China). Serum (collected without anticoagulant) was assessed with a fully automated biochemical analyzer (BC‐5130; Shenzhen Mindray Bio‐Medical Electronics Co. Ltd., China).

### 2.5 Histopatholog

After euthanasia, livers and kidneys were rapidly excised. Liver wet weights were measured after gentle blotting. Tissues were immersion‐fixed in 4% paraformaldehyde (0.1 M phosphate buffer, pH 7.4; 24 h, 4°C), dehydrated on an automatic processor (BMJ‐A, Changzhou suburb Zhongwei electronic instrument factory), embedded, sectioned, HE‐stained, and scanned on a Pannoramic 250 digital slide scanner (3DHISTECH, Hungary) for pathology evaluation. Lesions were graded 0–3 (none, mild, moderate, marked) using predefined quantitative criteria per INHAND/STP best practices; distribution (portal, mid‐zonal, centrilobular) was recorded (Colman et al. 2021). Two blinded readers scored all slides with consensus adjudication; inter‐reader agreement is reported for key endpoints.

### 2.6 Inflammation and Oxidative Stress

A portion of the liver tissue was homogenized for the detection of cytokines (interleukin‐4, IL‐4; interleukin‐6, IL‐6; interleukin‐10, IL‐10; tumor Necrosis Factor‐alpha, TNF‐α) and oxidative parameters (malondialdehyde, MDA; Hydrogen Peroxide, H_2_O_2_; reactive oxygen species, ROS; reactive nit rogen species, RNS). Cytokines were detected using an ELISA kit (Fine Test, Wuhan Fine Biotech Co. Ltd). Oxidative parameters were detected using chemiluminescence (Bestbio, Beijing Beibo Biotechnology). Follow the manufacturer's instructions to normalize the BCA (Beijing Solarbio Science & Technology Co. Ltd) content in the tissue.

### 2.7 Intestinal Microbiota

Fecal pellets (1–3 per mouse) were aseptically collected into sterile tubes immediately before terminal euthanasia. Genomic DNA was extracted using a commercial kit (TianGen Biotech, Beijing, China) following the manufacturer's protocols. The bacterial 16S rRNA V3‐V4 region was amplified with 314F (5′‐GGAAGTAAAAGTCGTAACAAGG‐3′) and 806R (5′‐GGACTACHVGGGTWTCTAAT‐3′) The fungal ITS1 region was amplified with ITS1F (5′‐GGAAGTAAAAGTCGTAACAAGG‐3′) and ITS2R (5′‐GCTGCGTTCTTCATCGATGC‐3′). Amplicons were purified for high‐throughput sequencing. Library preparation, sequencing, and primary bioinformatics were performed by Personal Biotechnology Co. Ltd. (Shanghai, China) on an Illumina NovaSeq platform (2 × 250 bp, paired‐end). Sequences were processed using either the QIIME2‐DADA2 denoising workflow (ASV inference) or the VSEARCH pipeline (OTU clustering). ASVs/OTU distributions were then used to assess within‐sample diversity, followed by downstream community analyzes. Differential abundance of metabolic pathways was evaluated in STAMP v2.1.3 using KEGG and COG annotations on median‐ratio‐normalized counts‐per‐million profiles.

### 2.8 Network Toxicological Analysis

Two databases, ADEMTlab 3.0 (https://admetlab3.scbdd.com/server/screening) and ProTox‐II 3.0 (https://tox.charite.de/protox3/index.php?site=compound_search_similarity), were used for in silico toxicological profiling of TEO (He et al. 2024). SMILES strings of the principal TEO constituents were retrieved from PubChem and submitted to both platforms to generate toxicity predictions, which were then downloaded for further analysis. For target prediction, the same SMILES strings were uploaded to the Swiss Target Prediction website (http://www.swisstargetprediction.ch/), ChEMBL (https://www.ebi.ac.uk/chembl/explore/targets/), and TargeNet (http://targetnet.scbdd.com/calcnet/index/). A probability threshold of > 0.1 was applied where applicable (Swiss Target Prediction, TargeNet) prior to exporting the predicted targets. We restricted searches on “ *Homo sapiens* ” aggregated the retrieved records, and evaluated structural consistency and data integrity. Datasets were then merged, de‐duplicated, and mapped to ChEMBL and STITCH identifiers. Target names were normalized according to UniProt conventions. The resulting, validated entries were integrated into a unified target database. In the final step, we built a comprehensive target database, integrating all the consolidated and validated targets. Disease‐associated targets were retrieved from GeneCards (http://www.genecards.org/) and OMIM (https://www.omim.org/) using the query “Liver Injury” with the species restricted to “ *Homo sapiens* ”. To ensure strong relevance, we applied a stringent selection threshold (GeneCards > 20) and retained genes meeting this criterion to construct a specialized liver‐injury target repository. The overlap between putative drug targets and disease targets was subsequently assessed using the Venny web tool (https://bioinfogp.cnb.csic.es/tools/venny/).

Protein–protein interaction (PPI) networks were generated using the STRING database (https://cn.string‐db.org/). Commonly obtained from the Venn analysis were uploaded to STRING with the species set to “ *Homo sapiens* ” to construct the PPI network. The resulting TSV file was exported for downstream analyzes; core (hub) targets were first screened in Excel and then evaluated with the NetworkAnalyzer plugin in Cytoscape v3.9.1. Targets with degree values ≥ 2× the median were defined as hubs. A drug–target–pathway network was subsequently assembled to visualize how TEO influences pathways via these targets, highlighting connections between key nodes and the compound to identify critical targets and associated pathways. Gene Ontology (GO: Biological Process, Molecular Function, Cellular Component) and KEGG pathway enrichment were performed using DAVID (https://david.ncifcrf.gov/), restricting species to *Homo sapiens* . The top 10 GO terms and top 20 KEGG pathways were selected based on *p*‐values (ranked from smallest to largest). Visualization of GO/KEGG results was carried out on the Microbiotics platform (http://www.bioinformatics.com.cn/).

### 2.9 Liver Metabolomics

Liver metabolomics (*n* = 6 per group) was conducted by Shanghai Personalbio Biotechnology Co. Ltd. After thawing, samples were vortexed (10 s) and spun (12,000 rpm, 5 min, 4°C). From each sample, 50 μL was reserved for protein quantification; another 50 μL was extracted with 250 μL 20% acetonitrile/methanol, vortexed (3 min), and centrifuged (12,000 rpm, 10 min, 4°C). The supernatant (250 μL) was chilled (−20°C, 30 min) and re‐centrifuged (12,000 rpm, 10 min, 4°C). A 180 μL aliquot of the final supernatant was subjected to LC–MS/MS (AB Sciex QTRAP 6500) for amino acid and metabolite profiling. Differential metabolites were identified by absolute log2 fold change (log2FC) between groups.

### 2.10 Statistical Analysis

Data is presented as mean ± standard error of the mean (SEM). Student's t test was applied for analysis between two groups. For multiple comparisons, we used one‐way ANOVA, followed by Tukey's multiple comparison test. *p* < 0.05 was accepted as statistically significant. Statistical analyzes were performed with GraphPad Prism 8 (GraphPad Software, La Jolla, CA, USA).

## 3 Results

### 3.1 Physiological and Biochemical Parameters

TEO showed no mortality orally at dosage levels of 1600 mg/kg. However, at 2900 mg/kg, one animal died, and at 5000 mg/kg, two animals died. Therefore, the acute oral LD_50_ (3800 mg/kg, mouse) is reported as an acute hazard indicator; it is not used to infer safety for intended use. Following oral administration of sub‐LD_50_ doses of TEO, mice exhibited a downward trend in body weight (Figure S1). Body weight remained essentially stable at 20 mg/kg, whereas higher doses were associated with progressive decreases. Liver mass also varied with dose, with an increase observed at 2000 mg/kg.

**FIGURE 1:**
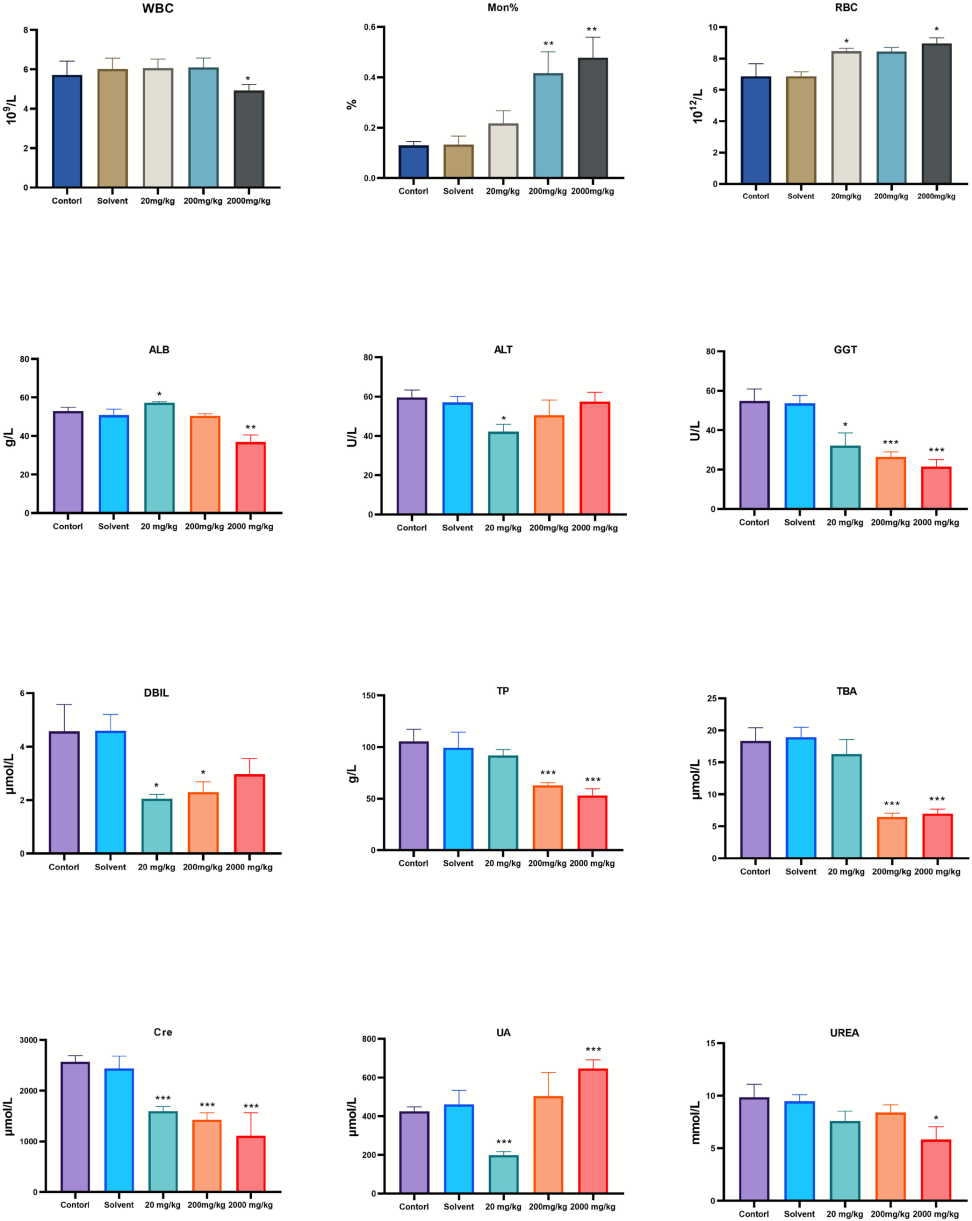
Differential change indicators between whole blood and serum. data are mean ± SEM (*n* = 6), *p* < 0.05 (*), *p* < 0.01 (**).

Meanwhile, we observed alterations in the hematologic profile. The solvent control and physiological saline groups showed comparable values across all indices. In whole blood, TEO treatment significantly affected WBC, Mon%, and RBC (Figure 1). A significant decrease in WBC was detected only at 2000 mg/kg, while Mon% increased significantly at 2000 mg/kg (with changes already evident at 200 mg/kg), and RBC increased beginning at 20 mg/kg. Other hematologic parameters were unaffected (Figure S2). By contrast, TEO exerted a greater impact on serum measures in mice. At doses ≥ 20 mg/kg, we observed reductions in ALB, GGT, TP, TBA, CRE, UREA, and elevations in ALT, DBIL, UA compared with the control group (Figure 1).

### 3.2 TEO Induces Histopathological Changes in the Liver and Kidneys of Mice

**FIGURE 2:**
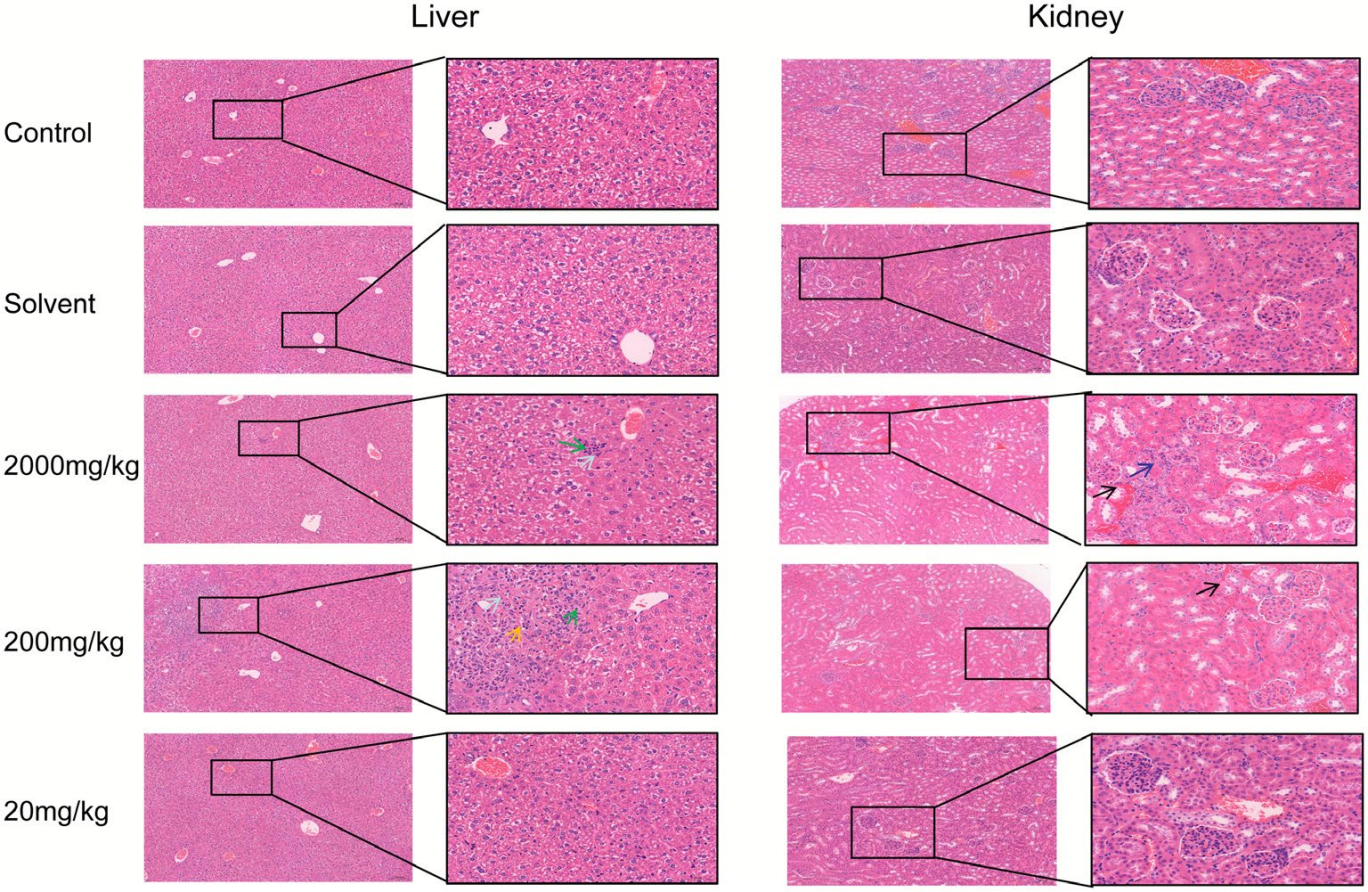
TEO induces pathological changes in the liver and kidneys of mice. Hepatocyte punctate necrosis (↑), neutrophils (↑), lymphocytes (↑), tubular epithelial cell vacuolar degeneration (↑), fibroblasts (↑). Control (physiological saline), Solvent (vehicle; 0.05% Tween‐80), 20 mg/kg (TEO 20 mg/kg), 200 mg/kg (TEO 200 mg/kg), 2000 mg/kg (TEO 2000 mg/kg).

Liver and kidney tissues from differently treated mice were fixed and H&E‐stained. In the control, solvent, and 20 mg/kg groups, liver architecture was preserved: central‐vein endothelium remained intact, hepatocytes were radially arranged around the central vein, and hepatic sinusoids appeared normal without notable congestion or inflammatory infiltration. Portal areas showed intact interlobular arteries, veins, and bile ducts, with no evident fibrous proliferation or periportal inflammatory cells. No significant pathological changes were observed in the liver. By contrast, in the 200 mg/kg group, focal hepatic necrosis was present, accompanied by perisinusoidal fibrosis. Spindle‐shaped fibroblasts with elongated ovoid nuclei were evident, and the necrotic foci showed mild hemorrhage with scattered red blood cells. At 200 and 2000 mg/kg, focal hepatocyte necrosis—ranging from spotty to confluent—was evident. Affected cells exhibited cytolysis with nuclear pyknosis/karyolysis, accompanied by mild neutrophilic infiltration (lobulated or band nuclei) (Figure 2).

In kidney tissues from the control, solvent, and 20 mg/kg groups, membranes were intact without evident connective‐tissue proliferation or inflammatory exudate. The corticomedullary junction was clear; cortical renal corpuscles were well defined with intact structures and a distinct Bowman's space. Renal tubules were largely preserved, and medullary collecting ducts were well formed without signs of degeneration, necrosis, or epithelial sloughing. No significant pathological changes were observed (Figure 2). By contrast, in the 200 and 2000 mg/kg groups, a small subset of tubular epithelial cells exhibited vacuolar degeneration with cellular swelling. In the 2000 mg/kg group, minimal interstitial fibrous proliferation was present, with fibroblasts displaying elongated ovoid nuclei.

### 3.3 Inflammation and Oxidative Stress

**FIGURE 3:**
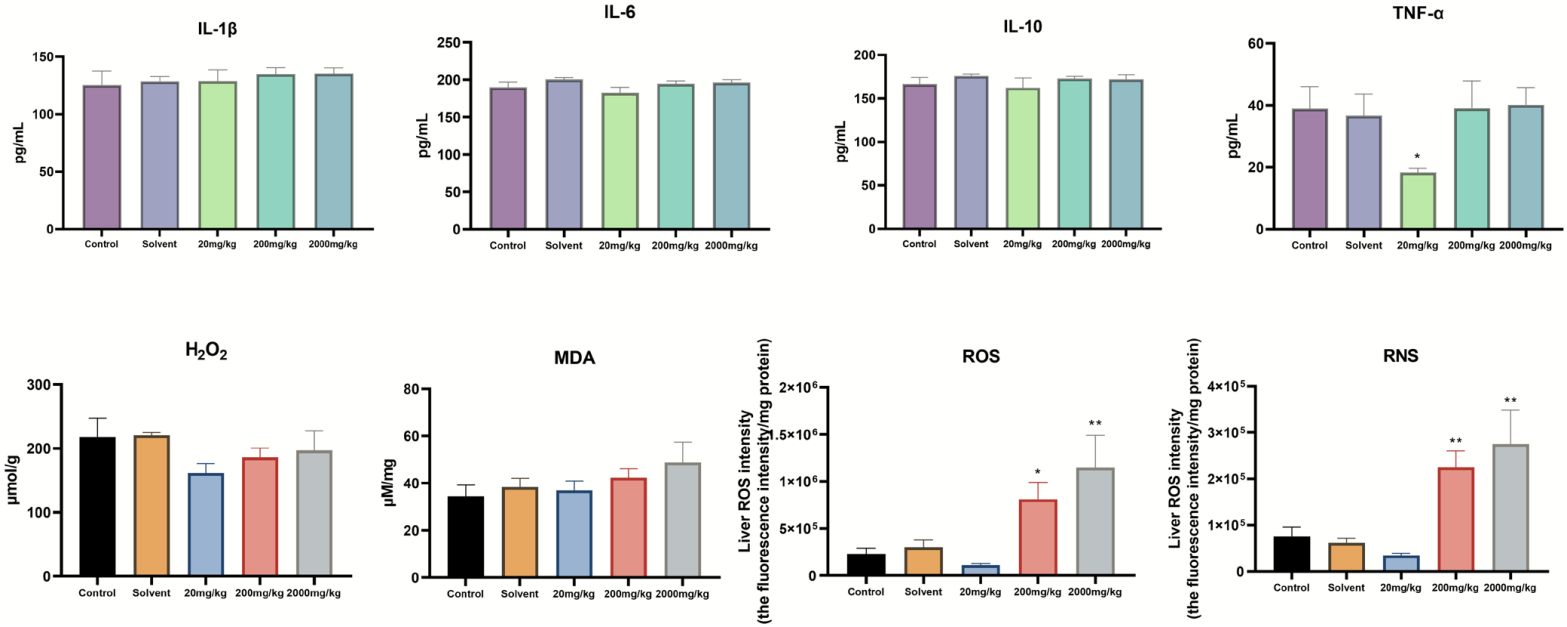
TEO modulates inflammatory cascades and oxidative stress markers in vivo. Control (physiological saline), Solvent (vehicle; 0.05% Tween‐80), 20 mg/kg (TEO 20 mg/kg), 200 mg/kg (TEO 200 mg/kg), 2000 mg/kg (TEO 2000 mg/kg). Data are mean ± SEM (*n* = 6), *p* < 0.05 (*), *p* < 0.01 (**).

Inflammatory factors and oxidative indicators in mouse liver tissues were detected using ELISA and chemiluminescence methods. Among the cytokines measured, only TNF‐α showed a significant difference compared to the control group at a dosage of 20 mg/kg (*p* < 0.05). The levels of hydrogen peroxide in the liver tissue were found to be lower than those in the control group, and they recovered with increasing doses of TEO. In contrast, MDA levels remained comparable to those of the control group and increased along with the dosage (Figure 3). The patterns of change for ROS and RNS in the liver tissue were similar. When the dosage of the essential oil reached 200 mg/kg, the levels of ROS and RNS in the liver showed a significant increase (*p* < 0.05), while a slight decrease was observed at the 20 mg/kg dosage.

### 3.4 The Effects of TEO on the Gut Microbiota of Mice

**FIGURE 4:**
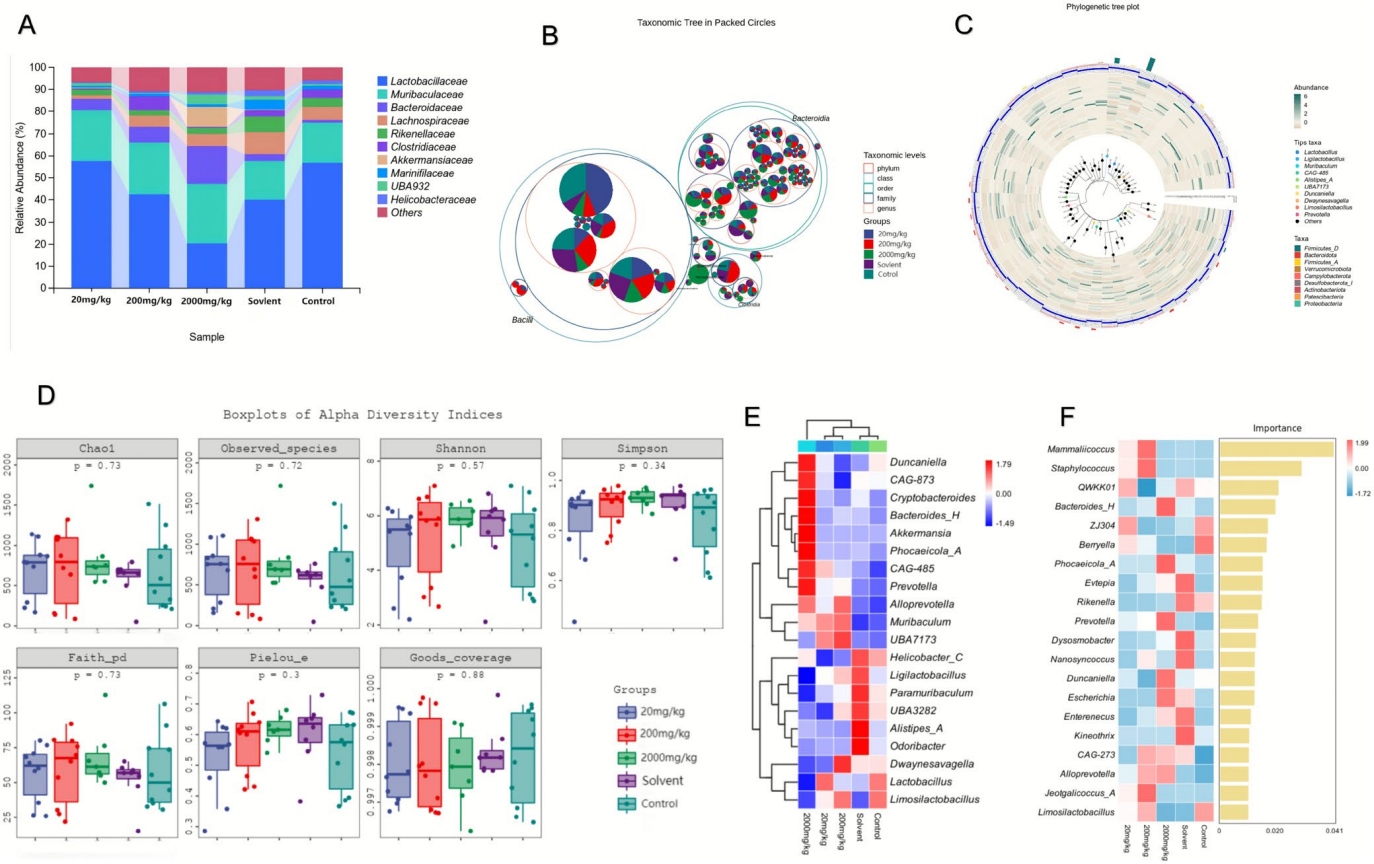
The effect of TEO on the gut microbiota composition in mice. (A, B) Analysis of gut microbiota composition in mice. (A) Phylum‐level and (B) genus‐level relative abundance stacked bars (colors are consistent across panels; see legend). Each bar represents one group. (C) Phylogenetic tree plot. Circular cladogram of detected taxa; highlighted clades indicate groups with higher median relative abundance (outer rings correspond to groups). (D) Alpha diversity analysis between different treatment groups of mice. Shannon, Observed species, Simpson, Chao1, Faith pd., Pielou e, and Goods coverage indices shown as box‐and‐whisker plots (median, IQR; whiskers 1.5 × IQR) with individual mice plotted as points. (E and F) Distribution of signature bacterial compositions. Control (physiological saline), Solvent (vehicle; 0.05% Tween‐80), 20 mg/kg (TEO 20 mg/kg), 200 mg/kg (TEO 200 mg/kg), 2000 mg/kg (TEO 2000 mg/kg). Data are mean ± SEM (*n* = 6), *p* or *q* < 0.05 (*), *p* or *q* < 0.01 (**).

Fecal microbiota analysis included both bacterial and fungal communities. The bacterial composition was broadly similar across groups, dominated by *Lactobacillaceae*, *Muribaculaceae*, *Rikenellaceae*, and *Bacteroidaceae*. In the 2000 mg/kg group, however, the relative abundances of *Clostridiaceae*, *Muribaculaceae*, and *Akkermansiaceae* were elevated, whereas *Lactobacillaceae* abundance was comparatively reduced (Figure 4A). Taxonomic classification further showed that *Bacteroidia*, *Bacilli*, and *Clostridia* represented the most abundant classes overall, while Verrucomicrobiota appeared exclusively in the 2000 mg/kg group (Figure 4B,C). However, the analysis of bacterial diversity among these different groups showed no significant differences in species richness, evenness, and diversity (Figure 4D). Through the analysis of differentially abundant bacteria, the marker species identified for the 2000 mg/kg group were *Duncaniella*, *CAG‐873*, *Cryptobacteroides*, *Bacteroides*, *Akkermansia*, *Phocaeicola*, *ACAG‐485*, *Prevotella*, and *Alloprevotella*. In contrast, the representative species for the TEO group included *Helicobacter*, *Ligilactobacillus*, *Paramuribaculum*, *UBA3282*, *Alistipes A*, *Odoribacter*, *Dwaynesavagella*, *Lactobacillus*, and *Limosilactobacillus*. Based on machine learning analysis, the marker species with the highest importance scores among these were *Mammaliicoccus*, *Staphylococcus*, *QWKKO1*, *Bacteroides H*, *ZJ304*, *Berryella*, *Phocaeicola A*, *Evtepia*, *Rikenella*, *Prevotella*, *Dysosmobacter*, and *Nanosyncoccus* (Figure 4E,F). Among these, the pathways with the highest abundance were Nucleoside and Nucleotide Biosynthesis and Amino Acid Biosynthesis. In addition, pathways related to Degradation/Utilization/Assimilation and Generation of Precursor Metabolite and Energy were also significantly enriched.

**FIGURE 5:**
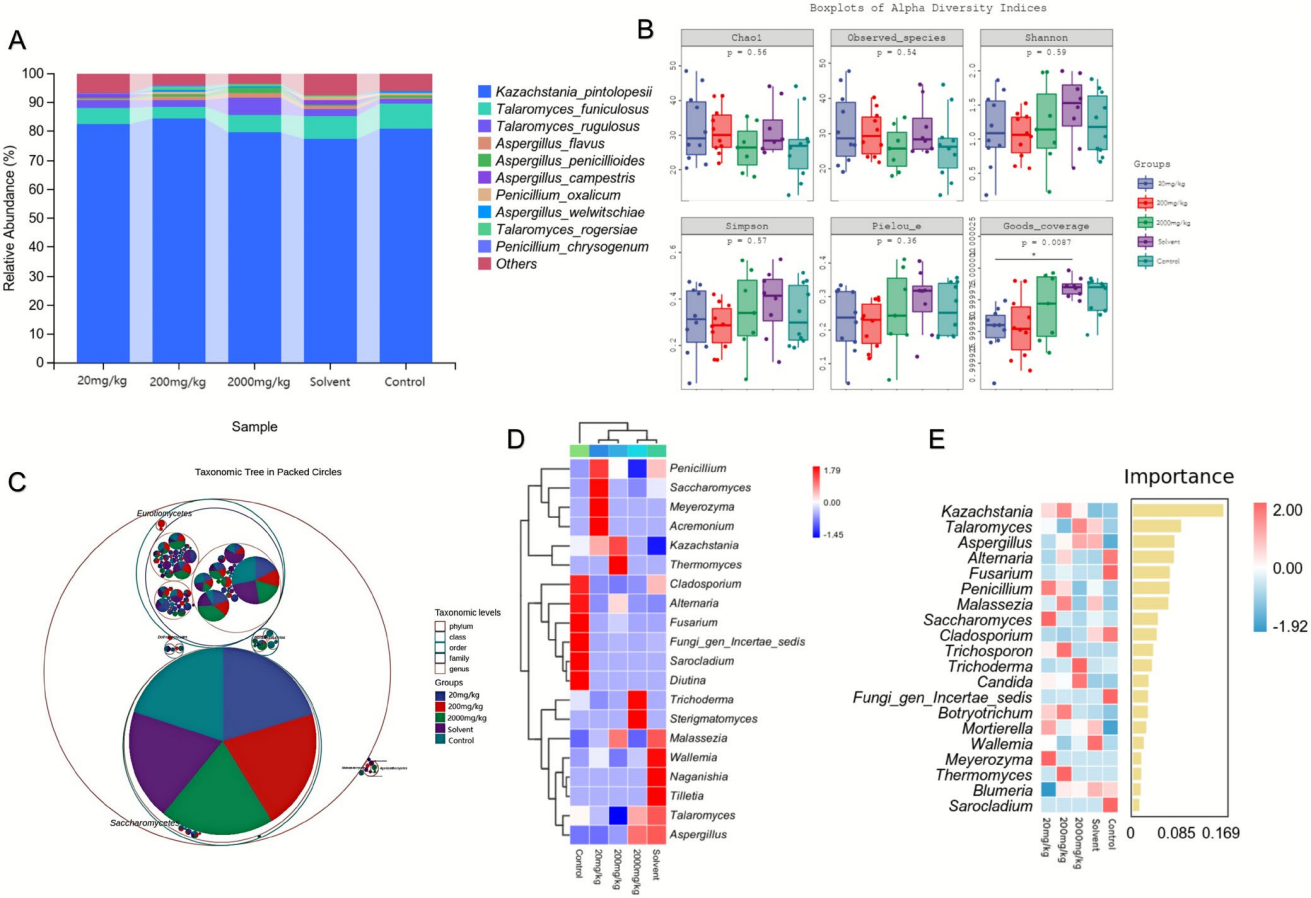
Effect of TEO on Fungal Composition in Mouse Gut. (A) Analysis of gut fungal composition in mice. Genus‐level relative abundance stacked bars (each bar = one group; colors consistent across panels; legend shown alongside). (B) Alpha diversity analysis between different treatment groups of mice. (C) Phylogenetic tree plot. (D and E) Distribution of signature fungal compositions, displayed as (D) volcano/forest plots and (E) grouped dot plots (per‐group values). Control (physiological saline), Solvent (vehicle; 0.05% Tween‐80), 20 mg/kg (TEO 20 mg/kg), 200 mg/kg (TEO 200 mg/kg), 2000 mg/kg (TEO 2000 mg/kg). Data are mean ± SEM (*n* = 6), *p* or *q* < 0.05 (*), *p* or *q* < 0.01 (**).

The same changes have also been observed in gut fungi. The fungal community composition was similar among the groups; however, there were differences in the relative abundance of species (Figure 5A). *Kazachstania pintolopesii* was the most abundant fungus, while *Talaromyces rugulosus* exhibited higher abundance in the TEO group. In contrast, *Talaromyces funiculosus* showed greater abundance in the control and solvent groups. Fungal composition analysis revealed minimal differences among the groups, primarily attributable to two distinct phyla (Figure 5B,C). Species composition analysis indicated that *Cladosporium*, *Alternaria*, *Fusarium*, *Fungi gen Incertae sedis*, *Sarocladium*, and *Diutina* were the primary populations exhibiting a decline in abundance following TEO treatment, while *Trichoderma*, *Sterigmatomyces*, *Kazachstania*, and *Thermomyces* demonstrated increased abundance. Random forest analysis identified *Kazachstania*, *Talaromyces*, *Aspergillus*, *Alternaria*, and *Fusarium* as the most important fungal taxa (Figure 5D,E).

### 3.5 Network Toxicity‐Based Analysis Predicted the Hepatotoxic Ingredients in TEO

**FIGURE 6:**
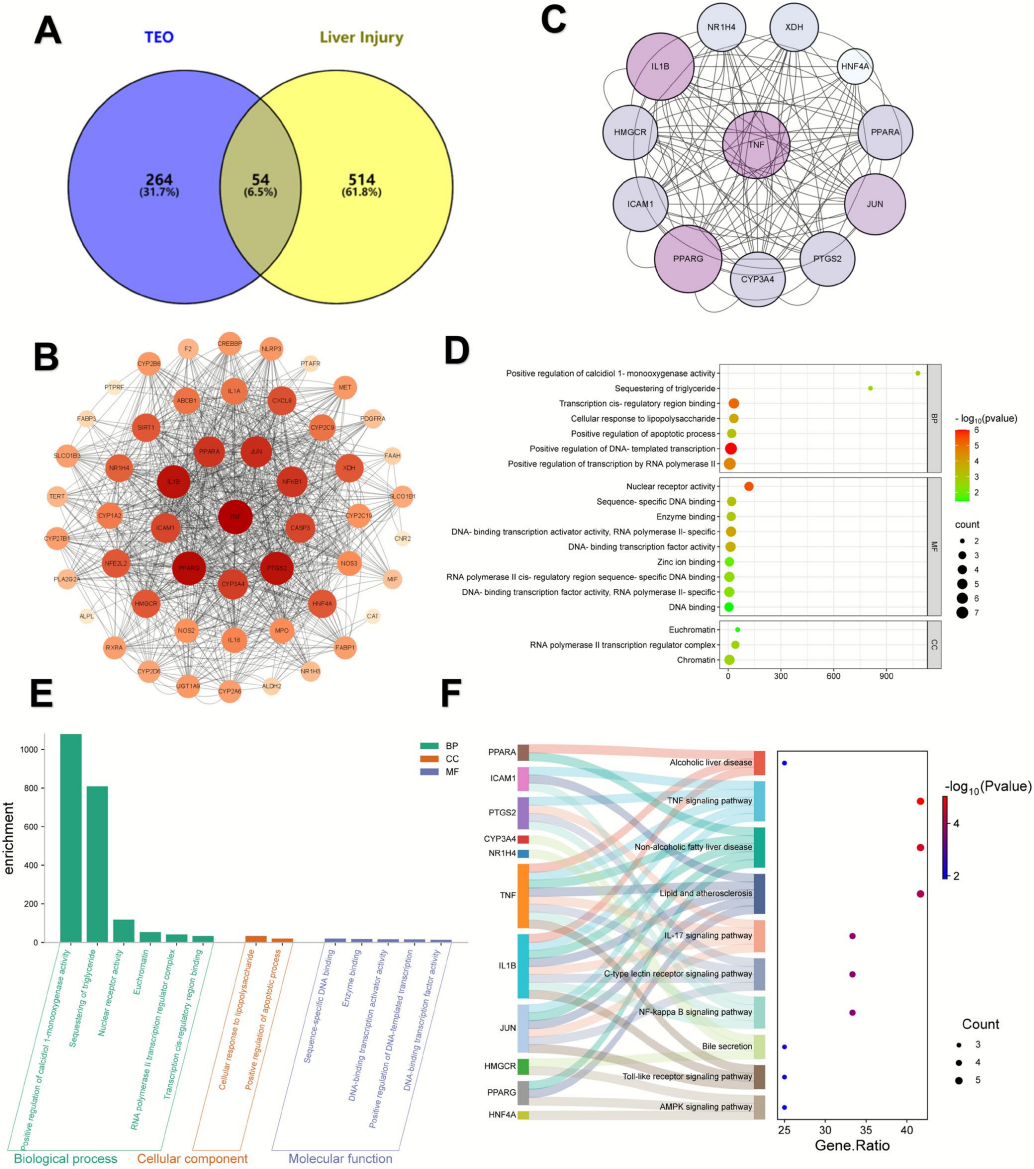
The network toxicology‐based discovery of hepatotoxic constituents and mechanistic hypothesis in TEO. (A) Venn diagram. Intersection between TEO‐predicted targets and hepatotoxicity‐related genes (numbers indicate set sizes; overlap denotes putative TEO–hepatotoxic targets). (B) The TEO hepatotoxic ingredient‐target network map. Edges: Predicted interactions. Node size is proportional to degree; color scale reflects constituent abundance. (C) Screen key targets of TEO‐induced hepatotoxicity. Top‐ranked targets identified by CytoNCA using degree ranking. (D–F) GO enrichment (Biological Process, Cellular component, and Molecular Function) (D, E) and KEGG pathways based on the STRING‐derived PPI network (F).

GC–MS analysis revealed 110 volatile constituents in TEO, representing 87.65% of the total oil. The major compounds included γ‐terpinene (33.38%), β‐myrcene (7.07%), and (E)‐3‐butylidene‐4,5‐dihydroisobenzofuran‐1(3H)‐one (5.71%). Terpenes (61 compounds) dominated the chemical profile, followed by fatty acids/esters (13) and alcohols (9) (Table 1). A total of 8 potential toxic ingredients and 318 action targets of TEO were summarized through the screening conditions detailed in the Materials and Methods section (Table 2). The targets associated with “liver injury” were screened from GeneCards and OMIM databases, resulting in 568 potential targets. Subsequently, a Venn diagram analysis identified 54 targets potentially induced by TEO in liver injury (Figure 6A). Next, the network diagram of “drug‐component‐target‐diseas” interaction was drawn via Cytoscape (Figure 6B), which was composed of 54 nodes and 854 associations. Subsequently, the core nodes of the network were filtered out via the CytoNCA plugin and the filtration conditions for 3 times were summarized in Figure 6C. Finally, the core network constituted of 12 nodes and 110 edges was built, comprising HMGCR, HNF4A, PTGS2, JUN, TNF, NR1H4, CYP3A4, XDH, PPARG, IL1B, ICAM1, and PPARA. Next, the vital ingredient targets of TEO were imported into DAVID for GO enrichment and KEGG pathway analysis. A total of 7 biological processes (BP), 3 cellular components (CC) and 9 molecular functions (MF) of TEO‐induced liver injury were obtained (Figure 6D,E). Among them, BP is mainly related to the positive regulation of calcidiol 1‐monooxygenase activity, sequestering of triglyceride, cellular response to lipopolysaccharide and positive regulation of apoptotic process; CC is mainly related to the centrosome, RNA polymerase II transcription regulator complex; MF is mainly related to nuclear receptor activity, sequence‐specific DNA binding, enzyme binding and RNA polymerase II cis‐regulatory region sequence‐specific DNA binding. To further analyze the hepatotoxic signaling pathway of TEO, KEGG enrichment analysis was performed, including alcoholic liver disease, TNF signaling pathway, non‐alcoholic fatty liver disease, lipid and atherosclerosis, IL‐17 signaling pathway, C‐type lectin receptor signaling pathway, NF‐κB signaling pathway, bile secretion, toll‐like receptor signaling pathway and AMPK signaling pathway (Figure 6E). GO and KEGG enrichment analyzes demonstrated that the potential hepatotoxic mechanism of TEO was thought to be involved in hepatocyte apoptosis, oxidative stress, inflammation and immunity in sum.

**TABLE 2:** Potential hepatotoxic constituents predicted by network toxicology.

Library	MF	CAS	SMILES
Phthalic anhydride	C_8_H_4_O_3_	85‐44‐9	C1=CC=C2C(=C1)C(=O)OC2=O
Butylated Hydroxytoluene	C_15_H_24_O	128‐37‐0	[2H]C([2H])([2H])C1=CC(=C(C(=C1)C(C)(C)C)O)C(C)(C)C
Z‐Butylidenephthalide	C_12_H_12_O_2_	72917‐31‐8	CCC/C=C\1/C2=CC=CC=C2C(=O)O1
Senkyunolide	C_12_H_16_O_2_	63038‐10‐8	CCCC [C@H]1C2=C(C=CCC2)C(=O)O1
(E)‐Ligustilide	C_12_H_14_O_2_	81944‐08‐3	CCC/C=C/1\C2=C (C=CCC2)C(=O)O1
trans‐13‐Octadecenoic acid, methyl ester	C_19_H_36_O_2_	1000333‐61‐3	CCCC/C=C/CCCCCCCCCCCC (=O) OC
Senkyunolide H	C_12_H_16_O_4_	94596‐27‐7	CCC/C=C\1/C2=C([C@@H] ([C@@H] (CC2)O)O)C(=O)O1
2,4‐dimethylbenzo[h]quinoline	C_15_H_13_N	605‐67‐4	CC1=CC(=NC2=C1C=CC3=CC=CC=C32)C

### 3.6 Analysis of Liver Metabolite Changes

**FIGURE 7:**
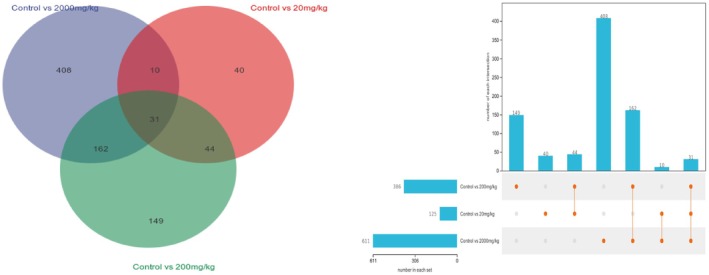
Number of differential metabolites between different dosage groups and the control group.

**FIGURE 8:**
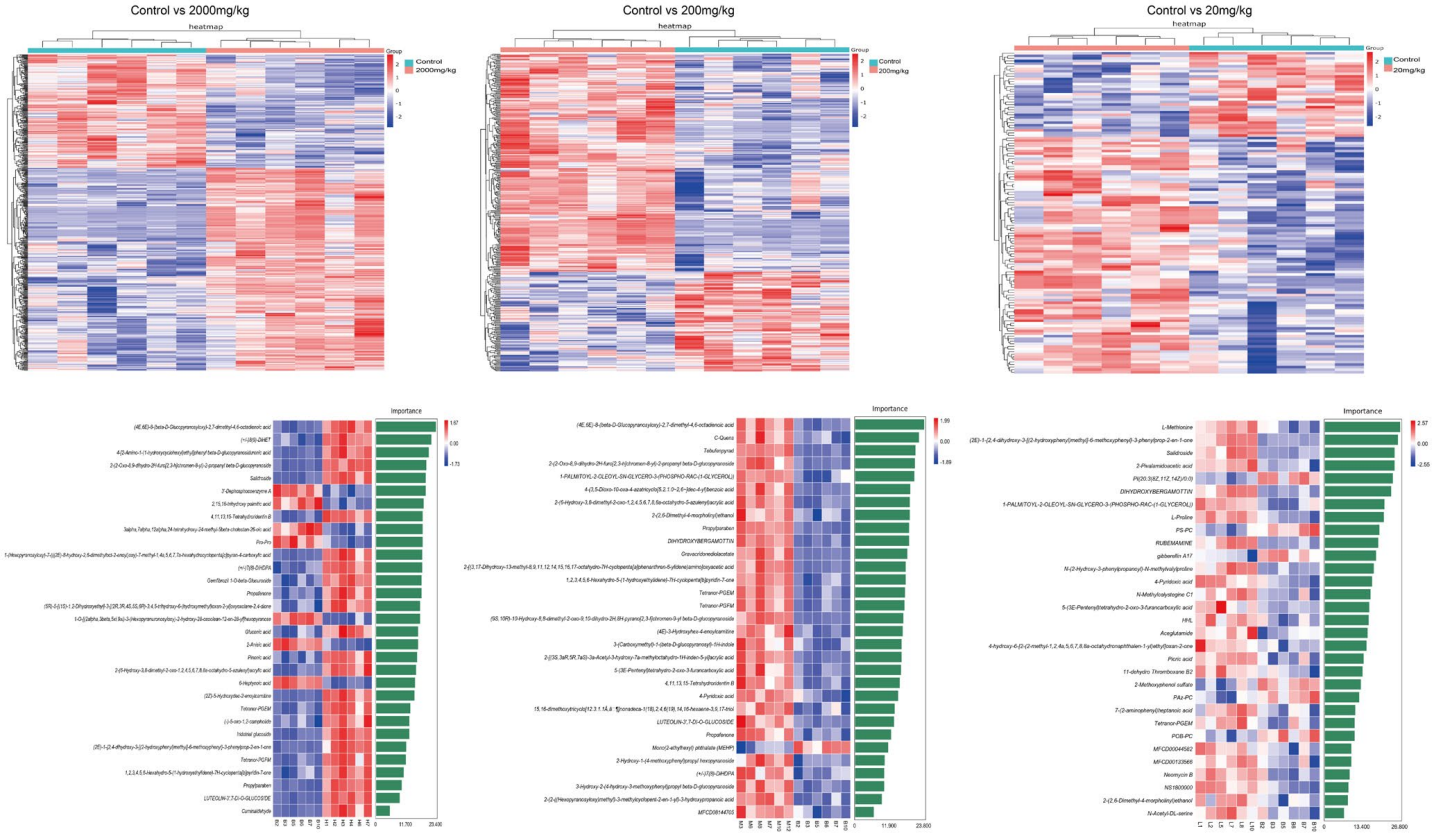
Differential metabolite analysis of liver in mice fed with TEO. Control (physiological saline), Solvent (vehicle; 0.05% Tween‐80), 20 mg/kg (TEO 20 mg/kg), 200 mg/kg (TEO 200 mg/kg), 2000 mg/kg (TEO 2000 mg/kg). Data are mean ± SEM (*n* = 6), *p* or *q* < 0.05 (*), *p* or *q* < 0.01 (**).

**FIGURE 9:**
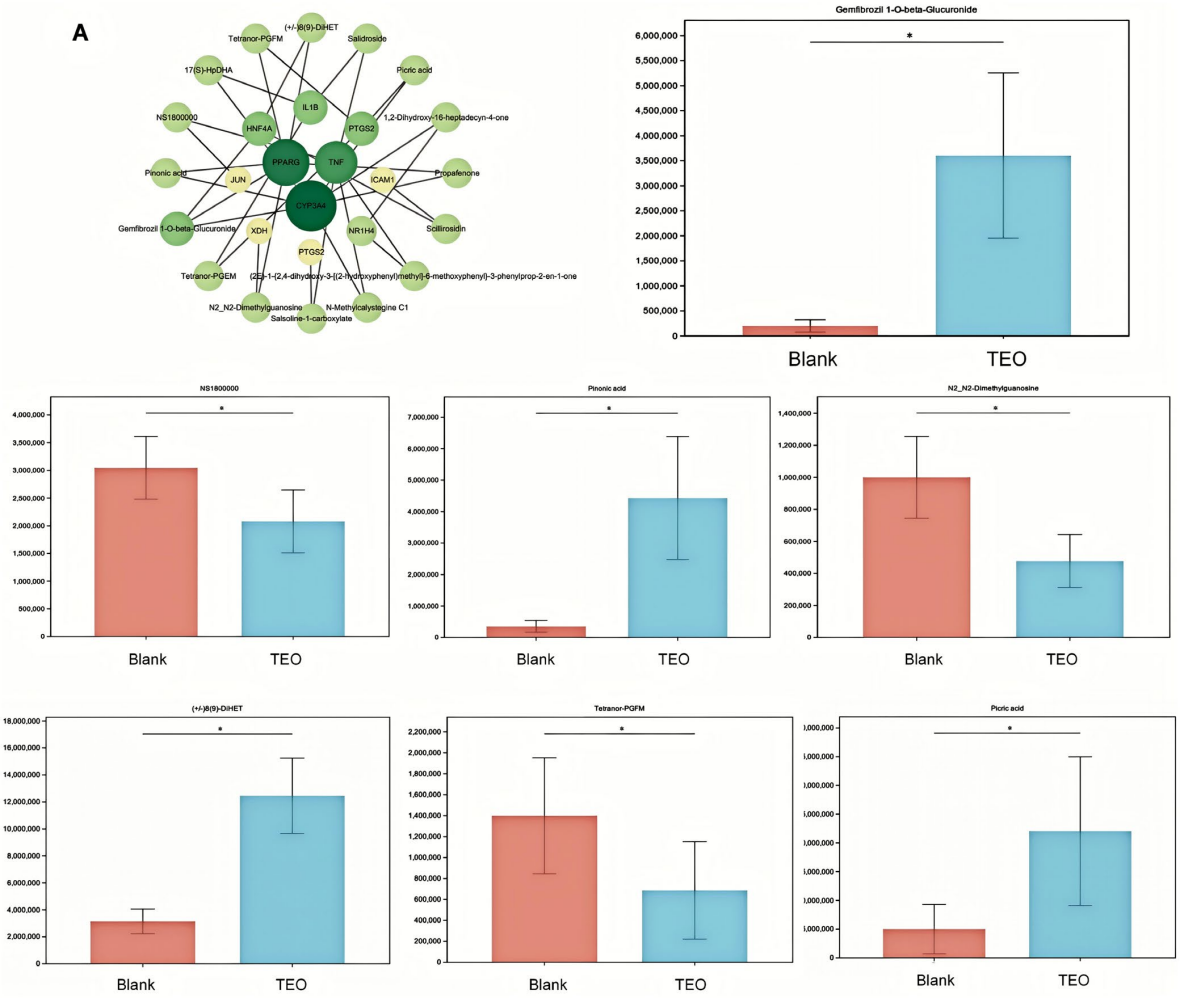
Linking liver metabolomics and network toxicology. (A) Differential metabolites and PPI network for screening potential targets in network toxicology. The network displays TEO constituents connected to overlapping hepatotoxicity targets. Node size is proportional to degree, and node color encodes cluster. Edges indicate predicted interactions. Built in Cytoscape using STRING PPI to guide topology. Differences in the metabolites Gemfibrozil 1‐O‐beta‐Glucuronide, Picric acid, Tetranor‐PGFM, Pinonic acid, NS1800000, (+/−)8 (9)‐DiHET, and DIHYDROXYBERGAMOTTIN between Control (physiological saline) and TEO (2000 mg/kg) groups. Data are mean ± SEM (*n* = 6), *p* < 0.05 (*).

Analysis of metabolites in the liver tissue of mice from different treatment groups was conducted and compared with the control group, resulting in a total of 844 differential metabolites. The 2000 mg/kg group had the highest number of differential metabolites, with a total of 611. Among these, 31 differential metabolites were found to be present in all three dosage groups (Figure 7). Pearson correlation coefficients were used to analyze the relationships among these differential metabolites, and machine learning analysis was performed to assess the importance of the differential substances (Figure 8). The results showed that metabolites such as Picric acid, Tetranor‐PGFM, Pinonic acid, NS1800000, (+/−)8 (9)‐DiHET, DIHYDROXYBERGAMOTTIN, and Gemfibrozil 1‐O‐beta‐Glucuronide exhibited differential expression (Figure 9). The metabolite‐related pathways were linked with key genes identified through network toxicology screening, and a “metabolite‐target” network diagram was constructed using Cytoscape (Figure 9A). Among them, PPARG, CYP3A4, and TNF were identified as the genes most strongly associated with metabolites, while Gemfibrozil 1‐O‐beta‐Glucuronide was the metabolite with the highest number of associated genes. Metabolomics has, to some extent, provided validation for the results of network toxicity.

## 4 Discussion

This study represents the first toxicological investigation of essential oil derived from the endangered plant *Thuja sutchuenensis*, establishing its LD_50_. Within the tested dosage range, TEO primarily induced oxidative stress in mice without causing inflammatory responses. Liver tissue metabolomics confirmed that TEO affects substance and energy metabolic pathways in liver cells. Additionally, gut microbiomics analysis revealed that TEO enhances gut microbiota diversity and increases the abundance of lactobacilli‐associated taxa.

Essential oils and plant extracts have been used for centuries in medicine, cuisine, perfumery, and cosmetics (Ferraz et al. 2022). Produced as secondary metabolites in response to stress, these volatiles function in plant defense and pollinator attraction (Radulović et al. 2017). As rich sources of bioactive compounds, they are drawing renewed interest amid consumer demand for natural, safer products and are now applied across food, agriculture, pharmaceuticals, cosmetics, and textiles. (Sattayakhom et al. 2023). Driven by rising consumer demand for natural products, industry adoption and research on essential oils are expanding. Within this context, *Thuja* species have attracted considerable interest owing to their distinctive aroma and application potential. Essential oils from *Thuja sutchuenensis*, *T. plicata* , and *T. orientalis* have shown diverse biological activities, notably antimicrobial and anti‐inflammatory effects, underscoring the substantial development potential of Thuja‐derived oils (Guleria et al. 2008; Han and Parker 2017; Pudełek et al. 2019; Swor et al. 2023). Nevertheless, even for natural products, rigorous safety evaluation remains indispensable (Sartori Tamburlin et al. 2021).

Acute toxicity testing was first used to delineate the upper hazard band of TEO in mice: doses above 2900 mg/kg threatened survival, and the oral LD₅₀ was about 3800 mg/kg. We emphasize that LD_50_ is a hazard indicator rather than a proof of safety for intended use; nonetheless, it usefully contextualizes our sub‐toxic work because it is orders of magnitude above doses commonly used in daily applications (Vora et al. 2024). Consistent with this framing, at sub‐LD₅₀ doses we observed no systemic inflammatory activation, yet detected metabolic alterations in liver‐centered readouts. These findings suggest that TEO's primary early impact under short‐term exposure is metabolic rather than overtly immunostimulatory. To avoid over‐interpretation, we refrain from inferring human safety from LD₅₀ alone; instead, we interpret the sub‐toxic changes as hypothesis‐generating and propose follow‐up repeat‐dose studies to define a no observed adverse effect level (NOAEL), convert to human‐equivalent doses, and derive margins of safety relative to realistic exposures (OECD 2020). These effects were similar to those reported for orange essential oil (Qu et al. 2023). In hematology, WBC count and NE% are commonly used indicators for systemic inflammation; however, TEO reduced WBC counts. This aligns with the cytokine data, indicating that TEO did not elicit an inflammatory response in mice. Additionally, biochemical markers such as ALT, AST, ALP, and GGT are closely associated with liver injury (Altmetric et al. 2022). In this study, ALP and AST showed no significant changes. Notably, GGT decreased in a dose‐dependent manner, whereas ALB and ALTexhibited dose‐specific responses. These findings suggest that mouse livers did not sustain severe injury, consistent with the histopathological findings. A similar pattern was observed for renal indices, where creatinine, urea, and uric acid fluctuated across groups; in particular, serum creatinine was significantly reduced in the TEO treated mice. We note that serum creatinine remains the most commonly used indicator of renal function in clinical practice and trials (Mercado et al. 2019; Ronco et al. 2019). However, serum creatinine concentration is influenced not only by renal excretion (filtration and tubular secretion) but also by creatinine synthesis, intake, and metabolism (Chaudhari and Hansen 2023). Low serum creatinine is considered a surrogate marker for muscle mass, potentially linked to the weight loss observed in the mice during the study. Histopathological examination showed no pathological changes in the kidneys, suggesting that TEO did not cause significant renal damage. This study was not designed to quantify systemic exposure; accordingly, we interpret our findings as acute hazard and early mechanistic signals. A dedicated OECD TG 417‐style toxicokinetic study with validated LC/GC–MS methods (FDA/EMA guidance) is planned to determine TEO constituent bioavailability and metabolism. A key limitation is the single post‐dose time point. Given the rapid disposition of volatile terpenes and the dynamics of inflammatory/oxidative pathways, earlier time points may exhibit different or transient patterns. Our findings should therefore be interpreted as a snapshot at 24 h post‐dosing, not a full temporal profile; a follow‐up time course (1 to 6 h, 24 to 72 h, 7 days) is planned to define onset, peak, and recovery.

Among these organs, the liver is the most vulnerable, being the primary site for endotoxin clearance. Therefore, evaluatin the toxicity of the products based on liver injury and metabolite changes is crucial (Li et al. 2016). Cytokine levels in liver tissue indicate that TEO did not elicit a hepatic inflammatory response; however, a significant rise in oxidative stress was observed. The predominant reactive species in biological systems are oxygen‐ and nitrogen‐derived‐ROS and RNS. ROS/RNS participate in key cellular processes, including proliferation, differentiation, migration, apoptosis, and necrosis. Maintaining low‐to‐moderate ROS/RNS levels is essential for physiological function, redox homeostasis, and the regulation of critical transcription factors (Jomova et al. 2023). Hydrogen peroxide is crucial for signal transduction induced by growth factors, the maintenance of thiol redox homeostasis, and the functioning of mitochondria. It is a potent oxidizing agent and one of the sources of ROS (Andrés et al. 2022). The results indicate that the increase in ROS and RNS induced by cypress essential oil is not attributed to hydrogen peroxide or MDA. In the analysis of metabolite pathway enrichment, no alterations were observed in lipid‐related pathways. Similarly, metabolites associated with natural products were also significantly observed, including DiHET and (2E)‐1‐{2,4‐dihydroxy‐3‐[(2‐hydroxyphenyl)methyl]‐6‐methoxyphenyl}‐3‐phenylpropen‐2‐one. This is clearly related to the compositional constituents of the TEO, as GC–MS analysis revealed a significant presence of terpenes, alcohols, and ketones (Swor et al. 2023). It is recognized that most of the known essential oil reveal valuable biological activities, but some of them also demonstrate a toxic character. In contrast, the safety profile of TEO significantly exceeds that of several other essential oils, such as *M. officinalis* oil, which has been shown to induce various pathological changes in the stomach, duodenum, liver, and kidneys at doses exceeding 1000 mg/kg, indicating that *M. officinalis* oil possesses moderate toxicity (Stojanović et al. 2019). Although most are safe for human food and medical applications, there are monoterpene compounds that, in certain amounts or under particular circumstances (e.g., pregnancy), can cause serious disorders (Wojtunik‐Kulesza 2022). The neurotoxicity of α‐terpineol has been established, and it is also associated with the induction of hepatic oxidative stress, cytotoxicity, and genotoxic damage (Baldissera et al. 2017, 2016). Thujone has also been shown to induce behavioral changes in mice, increase mortality rates, result in organ detachment, and decrease the weights of various organs including the liver, spleen, thymus, kidneys, and lungs, as well as cause disturbances in hepatic and renal function (Siveen and Kuttan 2011). Studies conducted in mouse models indicate that dietary administration of pulegone at a dosage of 30,000 ppm leads to impaired liver function, as well as increased weights of the liver and kidneys (Alan Andersen 2006). In summary, the analysis of metabolic components suggests that the substance and energy metabolism in the mouse liver has undergone alterations. Network toxicology involves the interdisciplinary integration of various scientific fields such as bioinformatics, big data analysis, genomics, and other related technologies (Zhao, Du, et al. 2023).

Non‐monotonic dose–response (NMDR) relationships are recognized across toxicology and pharmacology, particularly for complex mixtures and pathways with opposing feedbacks (Vandenberg et al. 2012). The TEO is chemically diverse, and literature on essential oils/terpenes supports such mixture‐ and context‐dependent behaviors (Bunse et al. 2022). Consistent with EFSA/OECD discussions on NMDR, we therefore interpret these profiles cautiously, focusing on pathway coherence (oxidative/immune axes) rather than assuming proportionality with dose (Agathokleous and Calabrese 2019). As a practical step, we now report goodness‐of‐fit for monotonic vs. NMDR models (AIC) and note that additional intermediate doses and minimal toxicokinetic sampling are planned to discriminate kinetics‐ vs. biology‐driven NMDR (Wan et al. 2024). Taken together, the network toxicology predictions and untargeted liver metabolomics converge on a mechanistic hypothesis implicating a TNF/PPARγ–CYP3A–ROS axis in TEO‐related injury. Although we did not include targeted interventions or protein‐level assays and thus refrain from causal claims, the predicted pathways are supported by metabolomic signatures and phenotypic assessments, suggesting that TEO‐induced liver toxicity may be mediated through immune signaling and oxidative stress.

As our understanding of human gut microbiota deepens, alterations in substance and energy metabolism will directly or indirectly impact the composition of gut microbiota. Consequently, gut microbiota health has emerged as a significant focus within the field of toxicology. The intestinal microbiota or its components and metabolites can directly or indirectly participate in the regulation of local and systemic pathophysiological processes of the host, hence greatly impacting the health of the host (Collins et al. 2023; Hung et al. 2019; Vicentini et al. 2021). Numerous studies indicate that shifts in gut‐microbiota composition and diversity accompany disease states (Chassaing et al. 2022). In our study, overall community diversity in TEO‐treated mice remained unchanged, whereas we observed a relative enrichment of lactobacilli‐related taxa—notably *Ligilactobacillus*, *Lactobacillus*, and *Limosilactobacillus* (Chuandong et al. 2024; Ma et al. 2023). Because “probiotic” is a strain‐level designation, we refrain from probiotic claims and instead interpret these shifts as health‐associated *lactobacilli* enrichment. Consistent with prior work, reductions in lactobacilli can have been associated with altered microbial metabolites and aggravate intestinal disease (Zhao, Ding, et al. 2023). Meanwhile, microorganisms involved in intestinal substances metabolism assist in processing organic matter, cellulose, and fatty acids, as well as boosting immunity (*Paramuribaculum*, *Alistipes A*, *Odoribacter*, *Kazachstania*, and *Talaromyces*) (Fang et al. 2023; Kim et al. 2019; Liu et al. 2024; Méndez‐Líter et al. 2021; Messaoudene et al. 2022; Parker et al. 2020; Zhang et al. 2022). Overall, TEO enhanced the abundance of lactobacilli‐associated taxa in the gut microbiota of mice. These studies provide a solid foundation for developing TEO as a treatment for diseases related to gut microbiota dysbiosis. Additionally, they offer a reference dosage range for essential oil development and introduce new methodologies for toxicological research.

## 5 Conclusion

The results of this study indicate that the LD_50_ of TEO is 3800 mg/kg, and no significant acute or subacute toxicity was observed at a dosage of 2000 mg/kg. The effects of TEO on liver injury in mice are primarily attributed to oxidative stress and hepatocyte apoptosis. Gut microbiota also confirmed that TEO can enhance the abundance of probiotics. In summary, this study represents the first toxicity evaluation of the essential oil derived from the endangered plant *Thuja sutchuenensis*, confirming its safety. These studies provide a strong basis for the sustainable development and protection of *Thuja sutchuenensis*.

## Author Contributions

Conceptualization, Hongping Deng, Nana Long, Quan Yang; Data curation, Youwei Zuo; Formal analysis, Nana Long, Renxiu Yao; Funding acquisition, Hongping Deng, Youwei Zuo, Nana Long; Methodology, Jian Li; Project administration, Shiqi You, Xiao Zhang; Software, Yang Peng; Validation, Renxiu Yao; Writing – original draft, Nana Long; Writing – review and editing, Hongping Deng, Nana Long.

## Funding

This work was supported by the National Key Protected Wildlife and Plant Project of the Central Forestry Reform and Development Fund [grant numbers zlg2021‐cq20211210 and zlg2022‐cq20220907]; the General Program of the Chongqing Natural Science Foundation [grant numbers CSTB2024NSCQ‐MSX1297]; the school‐level fund of Chengdu Medical College [grant number CYZYB24‐08]; and the special project of Liyan Workshop Aesthetic Medicine Research Center of Chengdu Medical College [grant number 25YM09].

## Conflicts of Interest

The authors declare no conflicts of interest.

## Supporting information


**Figure S1:** Body weight and selected biochemical parameters of mice.
**Figure S2:** Blood analysis of the effects of TEO on mice.

## Data Availability

The data that support the findings of this study are available from the corresponding author upon reasonable request.
