# Supplementary material for: Integrative Toxicology Profiling of Thuja sutchuenensis Essential Oil: From LD50 Safety Assessment to Hepatic Apoptosis and Gut Microbiota Modulation
Source: Food Sci Nutr. 2025 Dec 6;13(12):e71296. doi: 10.1002/fsn3.71296 (PMC12681077; doi:10.1002/fsn3.71296)
Supplement: Supplementary file 1 — Figure S1: Body weight and selected biochemical parameters of mice. Figure S2: Blood analysis of the effects of TEO on mice. [file FSN3-13-e71296-s001.doc]

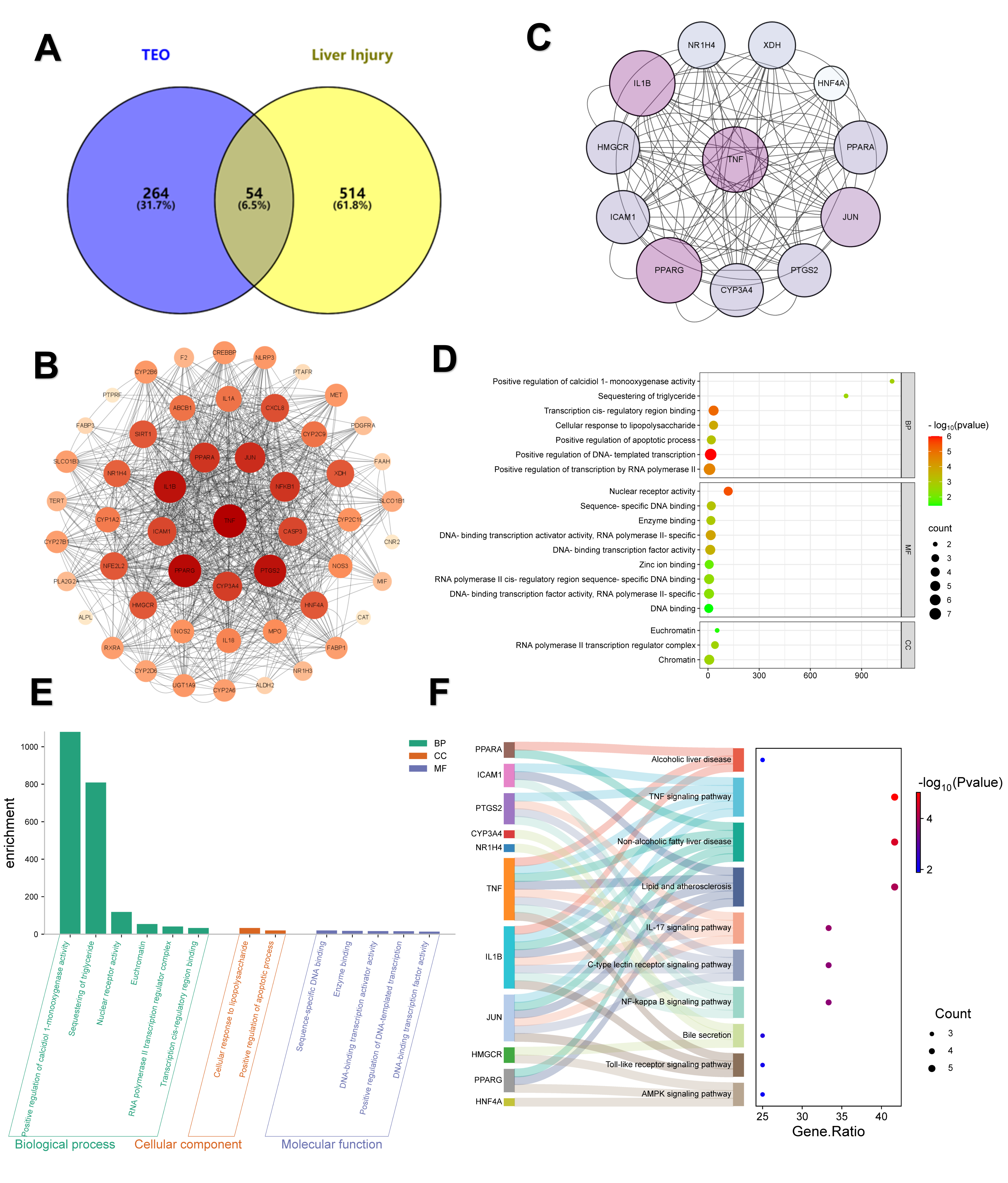

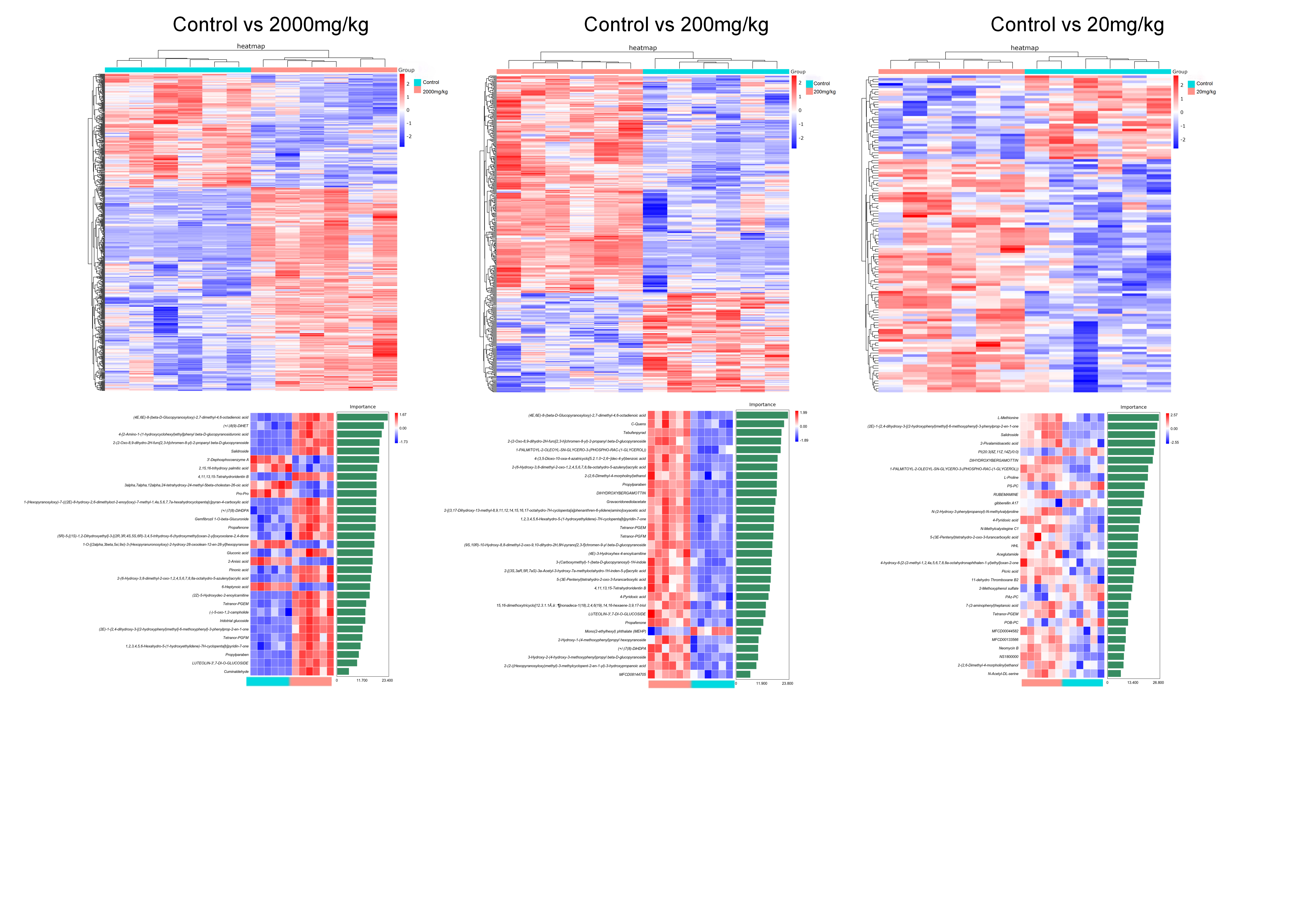

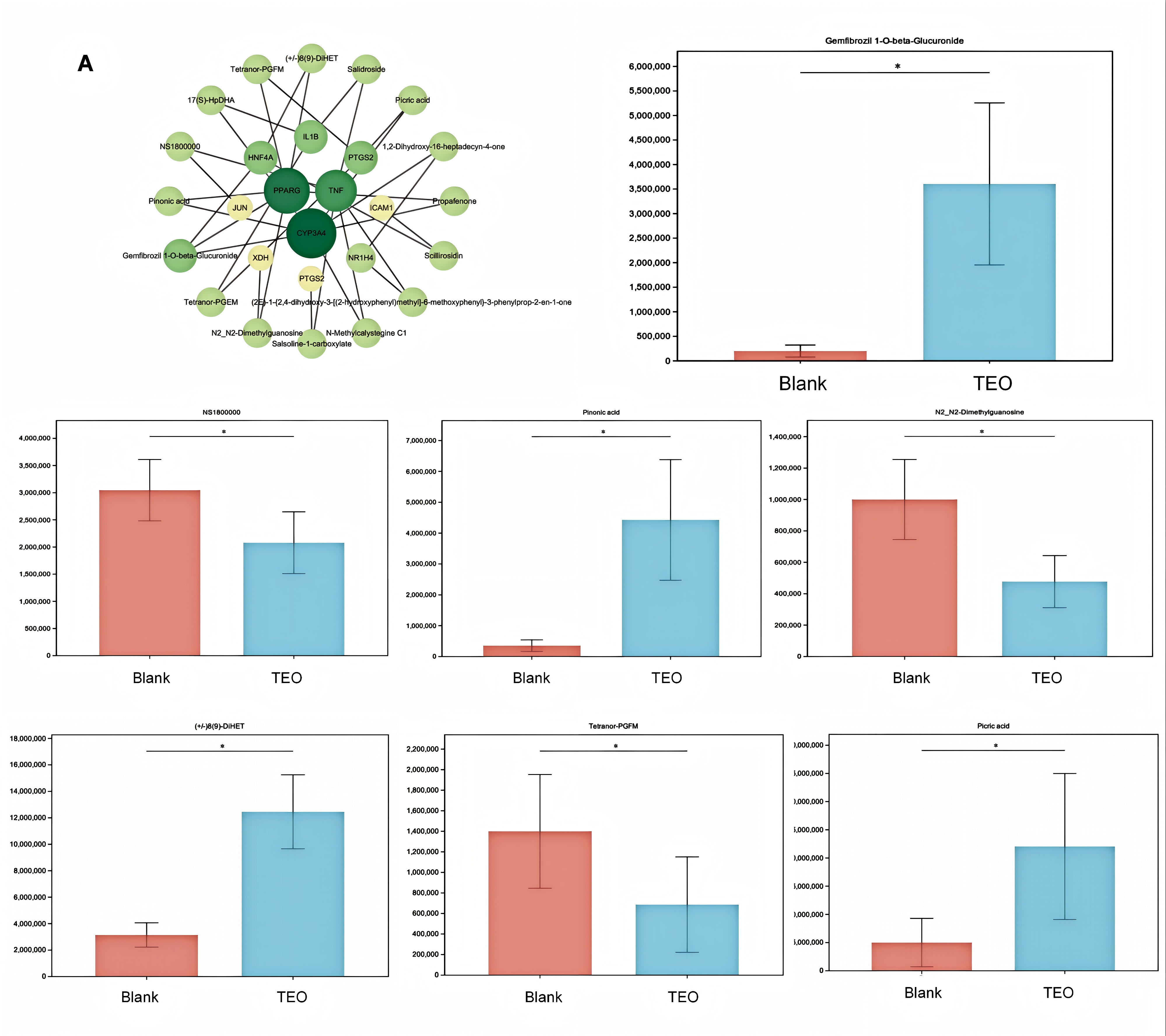


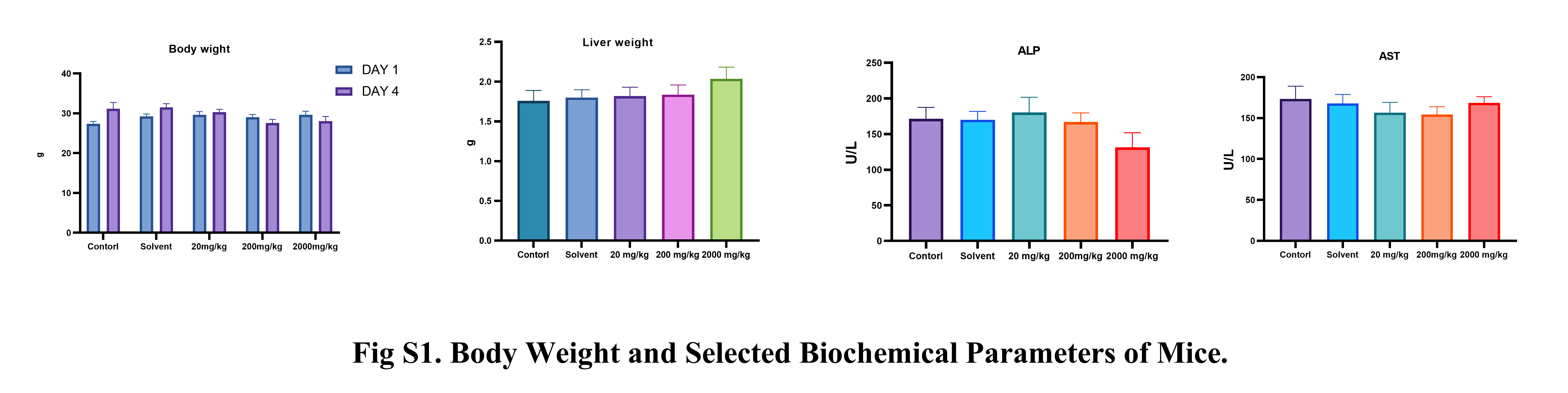
Figure S1. Body weight and selected biochemical parameters of mice.


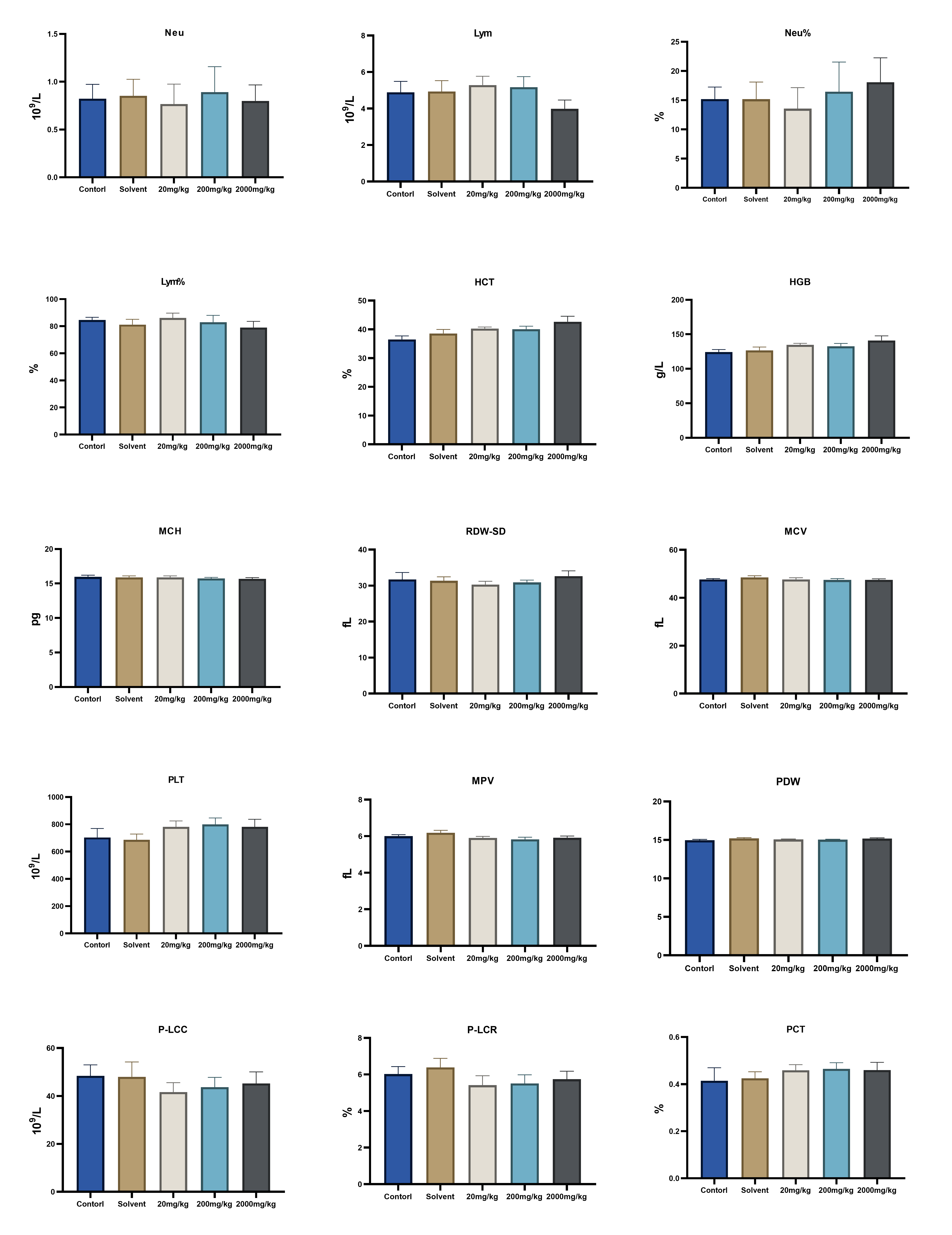
Figure S2. Blood analysis of the effects of TEO on mice.
